# Histone deacetylase 8 promotes innate antiviral immunity through deacetylation of RIG-I

**DOI:** 10.3389/fcimb.2024.1415695

**Published:** 2024-07-05

**Authors:** Huijun Zhang, Tingli Liu, Xinhua Liu, Fenfen You, Jiaheng Yang, Nan Zhang, Ying Huang, Gaofeng Liang

**Affiliations:** ^1^ Institute of Biomedical Research, Henan Academy Of Sciences, Zhengzhou, China; ^2^ School of Basic Medical and Forensic Medicine, Henan University of Science & Technology, Luoyang, China; ^3^ Department of Medical Laboratory, Fenyang College of Shanxi Medical University, Fenyang, China; ^4^ Pharmacy Department, Luohe Hosptial Of Traditional Chinese Medicine, Luohe, China

**Keywords:** HDAC8, antiviral, VSV, innate immunity, molecular mechanism

## Abstract

Histone deacetylates family proteins have been studied for their function in regulating viral replication by deacetylating non-histone proteins. RIG-I (Retinoic acid-inducible gene I) is a critical protein in RNA virus-induced innate antiviral signaling pathways. Our previous research showed that HDAC8 (histone deacetylase 8) involved in innate antiviral immune response, but the underlying mechanism during virus infection is still unclear. In this study, we showed that HDAC8 was involved in the regulation of vesicular stomatitis virus (VSV) replication. Over-expression of HDAC8 inhibited while knockdown promoted VSV replication. Further exploration demonstrated that HDAC8 interacted with and deacetylated RIG-I, which eventually lead to enhance innate antiviral immune response. Collectively, our data clearly demonstrated that HDAC8 inhibited VSV replication by promoting RIG-I mediated interferon production and downstream signaling pathway.

## 1 Introduction

Depend on the pattern recognition receptors (PRRs) which identify pathogen-associated molecular patterns (PAMPs), the innate immune system serves as the first line of defence against pathogen infections (Yoh et al., 2022). The RIG-I-like receptors (RLRs) detect viral RNA ligands or processed self RNA in the cytoplasm and induce antiviral innate immune responses (Loo and Gale, 2011). To date, RIG-I (retinoic acid-inducible gene I), MDA5 (melanoma differentiation-associated factor 5), and LGP2 (laboratory of genetics and physiology) are involved in RLRs signaling pathway (Saito et al., 2007). There are three distinct domains of RIG-I contained that C-terminal domain, central DExD/H helicase and N terminal which primarily detects viral 5-diphosphate or 5´-triphosphate of double-stranded RNA (dsRNA) and short dsRNA (Luo et al., 2011). Upon binding to its ligands, RIG-I undergo conformational changes and recruit MAVS (mitochondria antiviral signaling protein) through its CARDs (caspase activation and recruitment domain) (Ahmad et al., 2018). And then MAVS acts as a central platform for activating TBK1/IKKϵ kinases. Finally, transcription factors IRF3 is activated to drive type 1 interferon (IFN) production and antiviral gene expression (Rehwinkel and Gack, 2020).

Post-translational modifications, particularly deacetylation of key components, are critical for the regulation of the RLRs signaling pathway (Chang, 2021). Histone acetylation is a prevalent post-translational modification that regulate protein function, subcellular localization, and interactions (Tang et al., 2021). Histone deacetylases (HDACs, also named lysine deacetylases, KDACs), together with histone acetylases (HAT, also named lysine acetylases, KATs), were originally found to regulate gene expression by altering the structure of chromatin (Narita et al., 2019). To date, 18 human HDACs have been recognized that are grouped into four classes based on their sequence homology to yeast HDACs, class I (HDAC1-HDAC3, and HDAC8), class II (HDAC4-HDAC7, HDAC9, and HDAC10), class III (SIRT1 through SIRT7), class IV (HDAC11) (Qu et al., 2023). HDAC8 belongs to class I members of the HDAC family, there are many papers reported that HDAC8 involved in virus replication. For example, HDAC8 translocate from nuclear to cytosol and participate in viral processes in vaccinia virus infection (Oh and Broyles, 2005). Meng et al. showed that Rb, a well-known tumor suppressor, through HDAC1 and HDAC8 protects mice from RNA and DNA virus infection by suppressing IFN-β production (Meng et al., 2016). Another study showed that HDAC6 acts as a co-activator essential for enhancer activity, while HDAC1 and HDAC8 repress IFN-β expression (Nusinzon and Horvath, 2006). And Yamauchi et al. showed that HDAC8 could efficiently promote productive entry of influenza A virus in tissue culture cell and depletion of HDAC8 inhibited IAV infection (Yamauchi et al., 2011). However, another paper showed that miR-21–3p could promote influenza A virus replication by targeting HDAC8 and inhibiting its expression (Xia et al., 2018). HDAC6 regulates cellular viral RNA sensing by deacetylation of RIG-I (Choi et al., 2016). Another paper showed that HDAC6 activity is crucial for RIG-I-dependent signaling to innate antiviral immunity (Liu et al., 2016). More importantly, our previous study reported that HDAC8 in PK-15 or BHK-21 cell lines participated in the regulation of interferon-mediated immune response and inhibits the replication of FMDV (Foot-and-Mouth disease virus) (Zhang et al., 2023). However, the function of HDAC8 in immune response is far from understood and needs further exploration.

In this study, we demonstrated that VSV infection promotes the degradation of HDAC8 and knockout of HDAC8 in different cell lines significantly increases the replication of VSV. Mechanism study demonstrated that HDAC8 participated in the regulation of RIG-I mediated immune responses. To countered this effect, HDAC8 interacts with and promotes the deacetylation of RIG-I to facilitate antiviral response. Our data reported the interplay between HDAC8 and innate immune response, which provides a therapeutic intervention candidate for future study.

## 2 Materials and methods

### 2.1 Cells, viruses, and plasmids

Human embryonic kidney 293T cells, and HeLa cells were purchased from ATCC and maintained in Dulbecco’s modified Eagle’s medium (DMEM) supplemented with 10% fetal bovine serum (FBS) and 1% penicillin-streptomycin in a humidified incubator at 37°C with 5% CO_2_. HDAC8-Knockout (KO) cell lines were established using the CRISPR/Cas9 system following the published protocols. Two pairs of the small guide RNAs (sgRNA) targeting the HDAC8 gene of 293T cells and HeLa cells were designed using the CRISPR tool to establish the HDAC8-KO cell lines. Two sgRNAs were annealed and ligated to the pSpCas9 (BB) plasmid. The molecularly confirmed plasmids were transfected in 293T cells and HeLa cells, 3 μg puromycin was added per 1 ml of DMEM supplements, and the medium was renewed every 2 days. After puromycin selection, a single-cell clone was selected by the cloning ring anchoring method. The obtained monoclonal cell lines were identified by sequencing and Western blot. Vesicular stomatitis virus (VSV) and GFP-labelled VSV were proliferated amplified in 293T cells. pcDNA 3.1 plasmid was preserved in our Lab. HA-HDAC8 plasmid was obtained from Genecreate Company (Wuhan, China). HDAC8 knockout plasmids were constructed by subcloning the sgRNA (293T sgRNA TAGGTGGGAATATTACGATTGCGA, HeLa sgRNA GAAATTAATACGACTCA CTATAGATGCTGGACATACTTGACCGGTTTTAGAGCTAGAAATAGCAAG) into pSpCas9 -puro plasmid from Addgene (Watertown, Massachusetts, USA) following the protocol provided by Dr. Feng Zhang’s Lab.

### 2.2 Western blot analyses and immunoprecipitations

Cell precipitate was lysed in RIPA Lysis Buffer containing a 1% protease inhibitor cocktail. Total protein concentrations were quantified using the BCA Protein Assay kit obtained from Thermo scientific company. Equal amounts of protein samples were separated by sodium dodecyl sulfate-polyacrylamide gel electrophoresis (SDS-PAGE) and transferred to a polyvinylidene difluoride (PVDF) membrane. Then, the membrane was blocked with 5% skim milk at RT for 1 h, and the membranes were incubated with primary antibody overnight at 4°C. Western blot analyses were performed to detect the specific proteins with appropriate primary antibodies from different companies. Among them, HDAC8 (E7F5K), MAVS (D5A9E), RIG-I (D14G6), p-TBK1 (D52C2), TBK1 (E8I3G), p-IRF3 (E7J8G), and IRF3 (D6I4C), were purchased from Cell Signaling Technology (Shanghai, China). β-actin (T40104), GAPDH (M20006), Tubulin (M20005), HA (M10004), FLAG (M20008), GFP (7G9), were purchased from Abmart (Shanghai, China). After that, the membrane was washed with TBS-T buffer three times before incubation with HRP-conjugated secondary antibody for 1 h, followed by chemiluminescent detection.

### 2.3 Co-immunoprecipitation assay

HEK-293T cells were cultured in 60-mm dishes, and the monolayer cells were co-transfected with the indicated plasmids. The transfected cells were washed with PBS and lysed with 600 μl of lysis buffer. Anti-FLAG, anti-MYC, or anti-HA antibodies were used to immunoprecipitated the interacted proteins by 50%(v/v) slurry of GammaBind G Plus-Sepharose (GE Health Care Life Science, Piscataway, NJ, USA) overnight at 4°C. The precipitates were subjected to western blot.

### 2.4 Virus infection, RT-Qpcr, and TCID_50_ assay

293T cells and HeLa cells were cultured in 60-mm dishes to reach an approximate 90% confluence, which was washed once with PBS and incubated with VSV at a multiplicity of infection (MOI) of 0.1 at 37°C for 1h. Then, the cells were washed with PBS and cultured in 3 mL of FBS-free DMEM. After infection, the supernatant was removed, and 1 mL of TRIzol Reagent (Invitrogen, 15596026CN) was added to each dish. Total RNA was isolated according to the manufacturer’s instructions. RNA (1 μg) was used as the template for cDNA synthesis using PrimeScriptTM RT reagent Kit with gDNA Eraser (TAKARA, 639549). cDNA was then subjected to real-team PCR quantification using SYBR green Premix Ex Taq II (TAKARA, RR650A). The GAPDH gene was used as an internal control. The primers used in the experiment are listed as follow GAPDH F: GAGTCAACGGATTTGGTCGT, GAPDH R: GACAAGCTTCCCGTTCTCAG, VSV F: TGATAGTACCGGAGGATTGACGAC, VSV R: CCTTGCAGTGACATGACTGCTCTT. All the experiments were repeated at least three times, and relative mRNA expression levels were calculated using the threshold cycle (2-^△△^Ct) method. Viral titrations were determined using TCID_50_ assay. 293T cells were seeded in 96-well plates with 90% confluence, and a series of 10-fold serial dilutions from 10^-1^ to 10^-8^ of virus samples were prepared in another plate. One hundred microliters of the above samples were added to each well, and the plates were incubated at 37°C for 1 h. Then, the inoculum was removed, and cells were cultured in DMEM supplemented with 1% FBS for 72 h. All plates were analyzed by microscopic examination to determine the cytopathogenic effect (CPE).

### 2.5 Indirect immunofluorescence microscopy

The cells were seeded in NuncTM glass bottom dishes and cultured to a confluence of approximately 60–70%, then fixed with an acetone/methanol mixture (1:1) for 10 min at -20°C. 5% normal goat serum in PBS was used as the blocking buffer, and the fixed cells were washed with PBS and blocked for 1 h at 37°C. The anti-FLAG, anti-MYC or anti-HA primary antibodies were subsequently incubated overnight at 4°C. Cells were washed with PBS 5 times at room temperature (RT) (10 min each time). The fluorochrome-conjugated secondary antibodies were incubated with the cells in the dark for 1 h and washed with Tris-buffered saline (pH 7.6) three times at RT (10 min each time). The cells were incubated with 4’, 6-diamidino-2-phenylindole (DAPI, Roche, Diagnostics, Mannheim, Germany) for 10 min at RT to stain the nuclei. The stained cells and fluorescence were visualized using a Nikon eclipse 80i fluorescence microscope with appropriate settings. The microscopy images were processed using NIS Elements F 2.30 software.

### 2.6 Luciferase reporter gene assay

HEK293T cells (1×10^5^) were seeded on 24-well plates and transfected with the vector-control (100 ng), or HDAC8 (100 ng) and IFN-β luciferase reporter (100 ng) together with the renilla luciferase reporter plasmid pRL-TK (10 ng) with different dosage. At 36 h, luciferase activities were measured with Dual-Luciferase Reporter Assay System according to the manufacturer´s instructions. Data were normalized for transfection efficiency by dividing firefly luciferase activity by renilla luciferase activity.

### 2.7 Statistical analysis

This study’s quantified results were present as mean values ± s.e of three independent experiments. The student’s t-test was used to determine statistical significance. *p<0.05 was considered significant, and **p<0.01 was highly significant.

## 3 Results

### 3.1 VSV-GFP infection induced HDAC8 degradation in a time and dose-dependent manner

**Figure 1:**
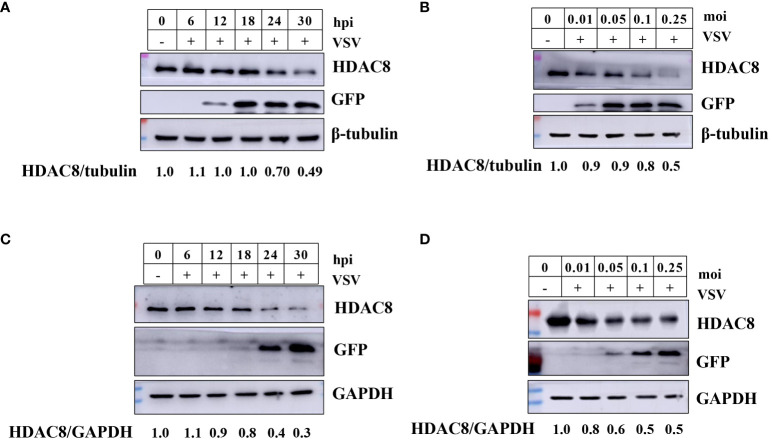
VSV-GFP infection decreases HDAC8 protein. **(A)** 293T cells were infected with VSV-GFP (0.1MOI) for 0, 6, 12, 18, 24, or 30 h, and endogenous HDAC8 and GFP proteins was detected by Western blot. **(B)** 293T cells were mock-infected or infected with 0.01, 0.05, 0.1, or 0.25 MOI of VSV-GFP at 24 h. Endogenous HDAC8 and GFP proteins was detected by Western blot. **(C)** HeLa cells were infected with VSV-GFP (0.1MOI) for 0, 6, 12, 18, 24, or 30 h, and endogenous HDAC8 and GFP proteins were detected by western blot. **(D)** HeLa cells were mock-infected or infected with 0.01, 0.05, 0.1, or 0.25 MOI of VSV-GFP at 24 h. Endogenous HDAC8 and GFP proteins were detected by western blot. All the Western blot data were repeated three times.

Our previous reports have shown that FMDV infection induced HDAC8 degradation in a time and dose-dependent. And now, we explored the change of HDAC8 during VSV-GFP infection by Western-blot. As shown in **Figures 1A, B**, in 293T cells, with the increasing infection time and multiplicity of infection, HDAC8 protein expression level decreased gradually. Similar data were also obtained in HeLa cells, VSV-GFP infection induced the decreased HDAC8 protein (**Figures 1C, D**). GFP antibody was used to monitor viral replication by Western blot. And the expression level of HDAC8 was normalized with β-tubulin or GAPDH. From the above results, we speculated that HDAC8 might involve VSV-GFP replication.

### 3.2 Knockout of HDAC8 increased VSV-GFP replication in 293T and HeLa cells

**Figure 2:**
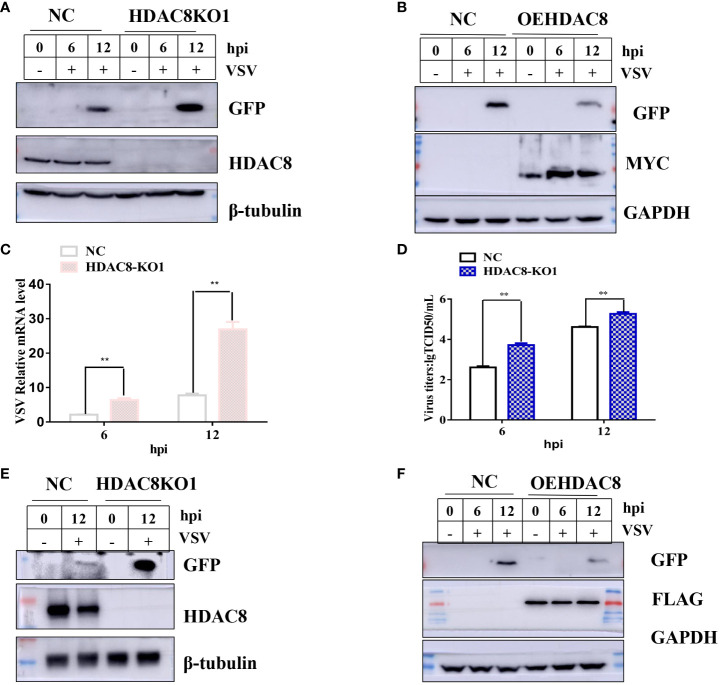
HDAC8 dysfunction promotes VSV-GFP replication. **(A)** NC and HDAC8-KO 293T cells were infected with VSV-GFP for 0, 6, 12 h, and the expression of GFP was detected by Western blot. **(B)** NC and OEHDAC8 293T cells were infected with VSV-GFP for 0, 6, or 12 h, and the expression of GFP was detected by Western blot. **(C)** NC and HDAC8-KO 293T cells were infected with VSV-GFP for 6 h and 12 h, and the expression of mRNA was detected by qPCR. **(D)** NC and HDAC8-KO 293T cells were infected with VSV-GFP for 6 h and 12 h, and the expression of titers was detected by TCID50 assay. **(E)** NC and HDAC8-KO HeLa cells were infected with VSV-GFP for 0, 12 h, and the expression of GFP was detected by Western blot. **(F)** NC and OEHDAC8 HeLa cells were infected with VSV-GFP for 0, 6, or 12 h, and the expression of GFP was detected by Western blot. All the Western blot data were repeated three times. *p<0.05; **p<0.01.

To further confirm our results, CRISPR/Cas9 technique was employed to knockout the *HDAC8* gene in two cell lines (293T and Hela cell lines). As shown in **Figures 2A, C**, compared with negative control cells (NC, which transfected empty plasmid), the *HDAC8* knockout cell lines showed no HDAC8 protein expression in 293T and Hela cell lines, which clearly shows the success of knockout of HDAC8 protein expression. The genome sequencing results are also consistent with the Western blot results. In 293T cells, clone 1 has eleven nucleotide deletion in the second exon, this lead to the early termination of protein translation and the genome sequencing results are also consistent with the Western blot results (**Supplementary Figures S1A–C**). While in Hela cell lines, compared with wildtype HDAC8 gene, the knockout clone 1 has two nucleotides deletion in the second exon, clone 2 has five nucleotides deletion in the second exon and these also lead to the early termination of protein translation (**Supplementary Figures S2A–C**). Viral replication was quantified between control and knockout cells to test the effect of HDAC8 dysfunction. As shown in (**Figure 2**), VSV-GFP replication was measured by Western blot (**Figures 2A, B**), Real-time PCR (**Figure 2C**), and viral titration (**Figure 2D**), the result demonstrated that virus replication was increased in HDAC8 knockout cells compared with control cells and over-expression HDAC8 significantly inhibited the replication of VSV-GFP. In HeLa cells, similar data was also obtained (**Figures 2E, F**). Altogether, these data demonstrated that HDAC8 could inhibit VSV replication.

### 3.3 HDAC8 is involved in the activation of innate immune signaling pathways

**Figure 3:**
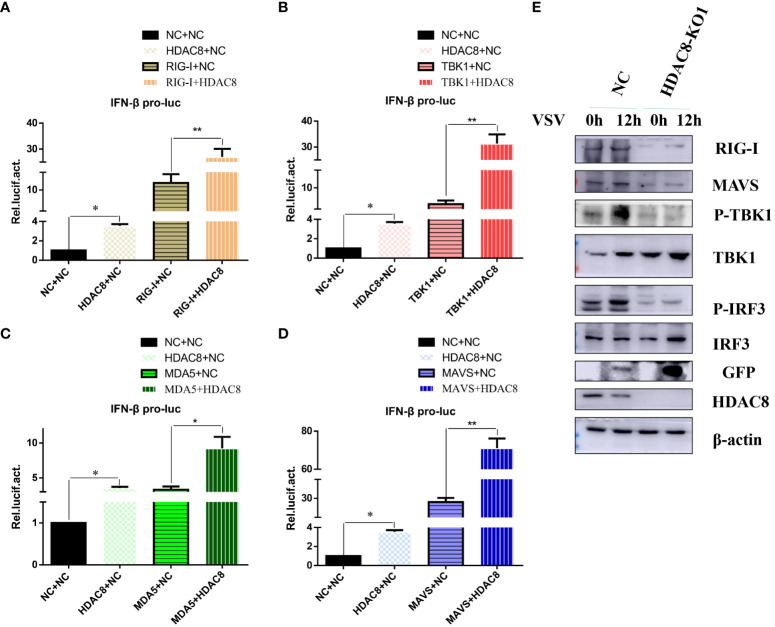
HDAC8 positively regulates RLRs signaling. **(A–D)** Effect of HDAC8 on the IFN-β promoter activity mediated by RLRs signaling pathway. HEK293T cells were plated in 24-well plates and transfected with the indicated combination. At 48 h post-transfection, the cells were used for luciferase activity assay. **(E)** NC and HDAC8-KO1 293T cells were seeded in 60-mm dishes and infected with VSV at an MOI of 0.1, and samples were collected at 12 h post-infection. The RIG-I, MAVS, p-TBK1, TBK1, p-IRF3, IRF3, GFP, HDAC8, and β-actin protein levels were detected by western blot. For luciferase assays were performed using a dual-specific luciferse asssay kit, data represented means ± SEM (n=3), and were tested for statistical significance. *p<0.05; **p<0.01.

Innate immunity is the first line of defense against virus infection. And we have showed that HDAC8 inhibited FMDV infection through positively regulate OAS, IFIT2, TNF, CCL5, and IFNB (Zhang et al., 2023). To identify the target of HDAC8 in RLRs signaling pathway, plasmids expressing key innate immune modulators (RIG-I, MDA5, MAVS and TBK1) were co-transfected with HDAC8 and the luciferase activity of IFN-β was determined. As shown in **Figure 3**. HDAC8 significantly promoted IFN-β activity stimulated by RIG-I (**Figure 3A**), TBK1 (**Figure 3B**), MDA5 (**Figure 3C**), and MAVS (**Figure 3D**). Because RIG-I primarily detects viral 5-diphosphate or 5´-triphosphate (5´ppp) of double-stranded RNA (dsRNA) and short dsRNA. In addition, Western blot results showed that compared with control cells, in 293T cell line knockout of *HDAC8* significantly reduced the protein expression of RIG-I, MAVS, p-TBK1 and p-IRF3 during VSV-GFP infection, which are key regulators of RLRs signaling pathway and subsequent immune response (**Figure 3E**). So, we speculate that the possible target of HDAC8 should be RIG-I or MDA5. Taken together, these results indicated that HDAC8 was involved in the innate immune pathway activation.

### 3.4 HDAC8 interacts with and deacetylates RIG-I

**Figure 4:**
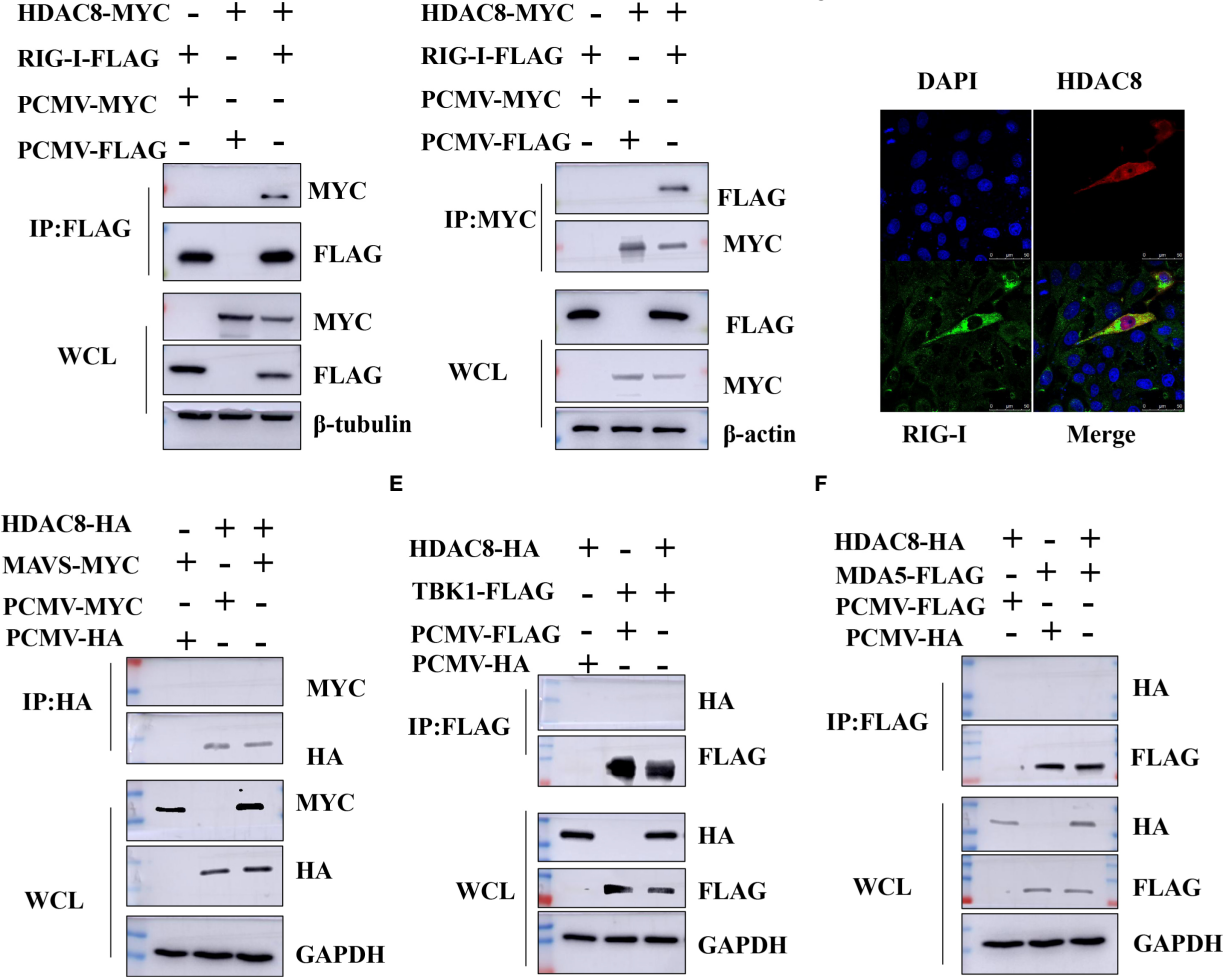
HDAC8 interacts with RIG-I but not MDA5, MAVS or TBK1. **(A, B)** HEK293T cells were transfected with HDAC8-MYC, RIG-I-FLAG or vector-control plasmids as indicated. At 24 h, cells were collected and Co-IP was performed with anti-MYC or anti-FLAG antibody. The WCL and Co-IP expression level of indicated proteins were detected by Western blotting. **(C)** HEK293T cells were co-transfected with FLAG-HDAC8 plasmids or empty vector and RIG-I-HA plasmid for 24 h. Cells were incubated with anti-FLAG (red), anti-HA (green), and DAPI (blue) for immunofluorescence staining and analyzed by confocal microscopy. **(D–F)** HEK293T cells were transfected with the indicated plasmids, and cell lysates were immunoprecipitated with HA or FLAG antibodies, followed by a Western blot with indicated antibodies. The WCL expression level of indicated proteins were detected by Western blotting. All the Western blot data were repeated three times.

**Figure 5:**
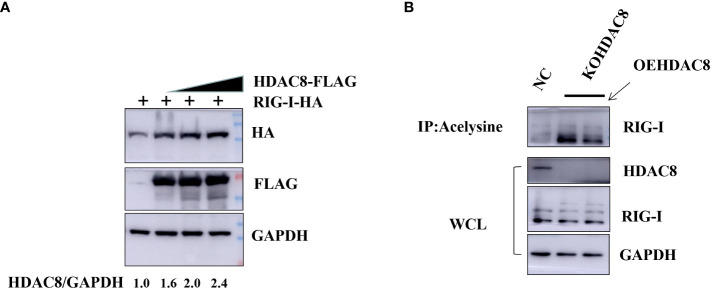
HDAC8 is responsible for augmenting and deacetylating RIG-I. **(A)** HEK293T cells were transfected with the increased amounts of HDAC8-FLAG plasmids (0, 1, 2 μg) and RIG-I-HA for 24 h. The expression level of total RIG-I, HDAC8 and GAPDH proteins were detected by Western blotting. **(B)** 293T-NC, 293T-KOHDAC8 and 293T-KOHDAC8 reconstituted with HDAC8 were subject to immunoprecipitation using anti-acelysine antibody and immunoblotted with anti-RIG-I, HDAC8 and GAPDH. All the Western blot data were repeated three times.

Previous data showed that HDAC8 through RIG-I, MDA5, MAVS and TBK1 promotes the IFN-β activity and regulates the host innate immune response. To confirm the interaction of HDAC8 with RIG-I protein, we examined whether HDAC8 binds to RIG-I by IP-western analysis after overexpressing HDAC8-MYC, RIG-I-FLAG or control vector for 24 h in transiently transfected HEK293T cells. After immunoprecipitated with anti-FLAG antibody, we could detect HDAC8 with anti-MYC antibody (**Figure 4A**), and immunoprecipitated with anti-MYC antibody, RIG-I was detected by western-blot (**Figure 4B**). Further immunofluorescence data also showed the colocalization of HDAC8 and RIG-I (**Figure 4C**). To examine the interaction of HDAC8 with the other key regulators of RLRs signaling pathway such as MDA5, MAVS or TBK1, we examined whether HDAC8 binds to MAVS, TBK1 or MDA5 by IP-western analysis after overexpressing HDAC8-HA and MAVS-MYC, TBK1-FLAG or MDA5-FLAG for 24 h in transiently transfected HEK293T cells. As shown in **Figures 4D–F**, immunoprecipitation experiments did not verify the interaction of HDAC8 with these three proteins MAVS (**Figure 4D**), TBK1 (**Figure 4E**) or MDA5 (**Figure 4F**). The above results showed that HDAC8 interacting with RIG-I but not the other RLRs signaling pathway such as MAVS, TBK1 or MDA5. To further confirm the effect of HDAC8 in the RIG-I protein expression, we transiently transfected the expression plasmids of HDAC8-FLAG and RIG-I-HA. As shown in **Figure 5A**, HDAC8 significantly promoted the protein expression of RIG-I in a dose-dependent manner. So we speculate HDAC8 might be a deacetylase about RIG-I. As speculated, RIG-I was acetylated in HDAC8KO 293T and reconstituted HDAC8 successfully deacetylated RIG-I in HDAC8KO 293T, indicating that HDAC8 is a deacetylase for RIG-I (**Figure 5B**).

## 4 Discussion

**Figure 6:**
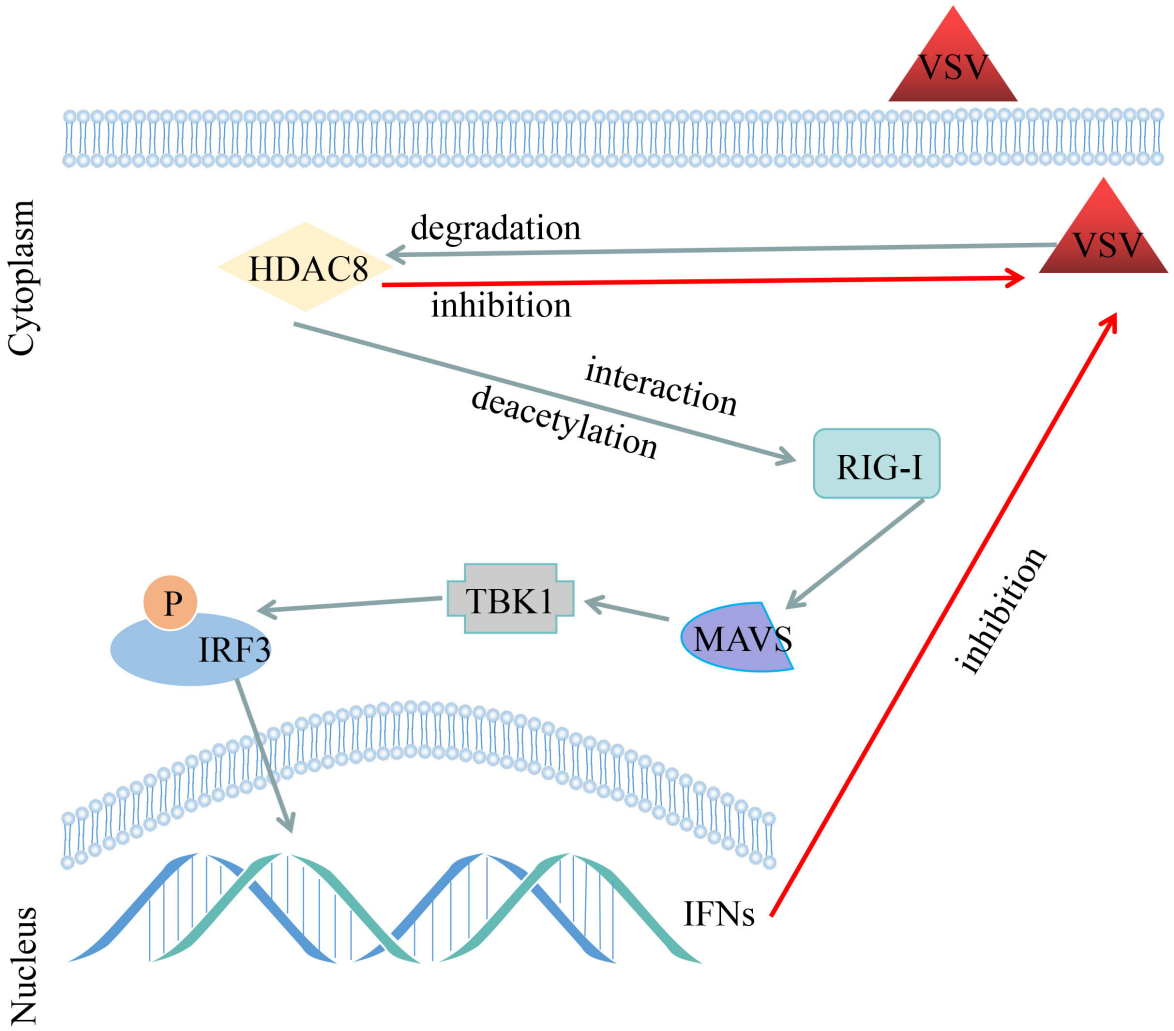
Schematic representation shows the model of VSV-GFP infection decreased the HDAC8 protein expression and HDAC8 interacts with and promotes the deacetylation level of RIG-I to promote the antiviral response.

Acetylation is one of the most important post-translation modifications of proteins (Feng et al., 2018). And a clear role of acetylation in the infection of viral which cause infections is emerging (Xue et al., 2022). So far, our lab have showed that knockout HDAC9 in BHK-21 cells increased FMDV replication and HDAC4 suppressed FOXO1 transcriptional activity to increase the interferon production and antiviral response (Ren et al., 2021; Hou et al., 2022). More importantly, our recent study showed that FMDV VP3 protein promoted autophagic degradation of HDAC8 and HDAC8 positively regulated the innate response (Zhang et al., 2023). However, the molecular mechanism of HDAC8 in the innate response remains to be elucidated. In this study, we have demonstrated that VSV infection significantly reduced the protein expression of HDAC8 with time and dose dependent in different cell lines. Secondly, knockout HDAC8 by using CRISPR/Cas9 technology significantly enhanced virus replication while over-expression HDAC8 present opposite results. Thirdly, HDAC8 involved in antiviral immune response and interacts with and deacetylates RIG-I which promoted interferon production and antiviral response (**Figure 6**).

RLR signaling plays an essential role in recognizing virus-derived RNAs in the cytoplasm (Zheng et al., 2023). Activation of RLR signaling results in an antiviral response necessary to suppress the spread of virus (Qin et al., 2023). Limiting excessive activation of RLR signaling is critical to protect the host from an unbalanced response and inflammatory injury (Qin et al., 2023). So far, there are many studies shown that HDACs are the regulator of signal transduction activation in an innate immune response.

Larguet et al. showed that HDAC1 interacts with the HIV-1 integrase and to facilitate HIV-1 replication with a effect on a preintegration step (Larguet et al., 2019). HDAC1 facilitated viral replication via two paths: promoting the nuclear retention of NP and inhibiting TBK-IRF3 pathway (Chen et al., 2017). While another reported showed that Influenza A virus dysregulates host histone deacetylase 1 that inhibits viral infection in lung epithelial cells (Nagesh and Husain, 2016). The reason about that may the cell lines is different in above results. Another paper showed that HDAC2, a class I member and closely related to HDAC1 in structure and function, also possesses anti-IAV properties (Nagesh et al., 2017). Guo et al. showed that genetic and pharmacological inhibition of HDAC1 significantly influenced PRV replication and mechanistically, the HDAC1 inhibition induced DNA damage response resulted in the release of double strand DNA into the cytosol to activate cyclic GMP-AMP synthase and the downstream STING/TBK1/IRF3 innate immune signaling pathway (Guo et al., 2021).

Xu et al. reported that HDAC1 negatively regulates porcine epidemic diarrhea virus replication, while PEDV utilizes its N protein to interact with Sp1 within the nuclei, halting its transcriptional activity of HDAC1 expression in favor of viral replication (Xu et al., 2021). HDAC9 directly maintains the deacetylation status of TBK1 and enhances its kinase activity to activate antiviral innate immunity (Li et al., 2016). And we previous studies showed that Knockout HDAC9 promoting the replication of FMDV (Hou et al., 2022). HDAC4 promotes IFN-I signaling and co-precipitate with STAT2 and restricts VSV and HSV-1 replication and spread. HDAC3 inhibitor treatment also blocked HCV replication in a mouse model of HCV infection (Lu et al., 2019). Tang et al. showed that HDAC3 promotes innate antiviral immunity through deacetylation of TBK1 (Tang et al., 2021). And another paper reported that the deubiquitinating and stabilizing HDAC3 by ATXN3 to positively regulates antiviral response (Feng et al., 2018). In addition, as a deacetylase, HDAC6 promotes innate antiviral immunity through deacetylates RIG-I to recognize and restrict RNA viral infection (Choi et al., 2016). And in this study, we showed that HDAC8 also could deacetylated RIG-I and promoted antiviral response.

In conclusion, our data showed that HDAC8 inhibited VSV replication by interacting with and deacetylating RIG-I; the decreased acetylation of RIG-I lead to higher interferon production and promote antiviral response. With the finding of more and more HDAC8 specific inhibitors, these findings provided a potential therapeutic strategy for the prevention and control of viral diseases.

## Data availability statement

The original contributions presented in the study are included in the article/**Supplementary Material**. Further inquiries can be directed to the corresponding authors.

## Author contributions

HZ: Conceptualization, Data curation, Methodology, Writing – original draft, Writing – review & editing. TL: Methodology, Writing – original draft. XL: Data curation, Writing – original draft. FY: Formal analysis, Writing – original draft. JY: Data curation, Writing – review & editing. NZ: Methodology, Supervision, Writing – original draft. YH: Data curation, Writing – original draft. GL: Conceptualization, Funding acquisition, Writing – review & editing.
