# Supplementary figures and images for: Histone deacetylase 8 promotes innate antiviral immunity through deacetylation of RIG-I
Source: Front Cell Infect Microbiol. 2024 Jul 5;14:1415695. doi: 10.3389/fcimb.2024.1415695 (PMC11257846; doi:10.3389/fcimb.2024.1415695)

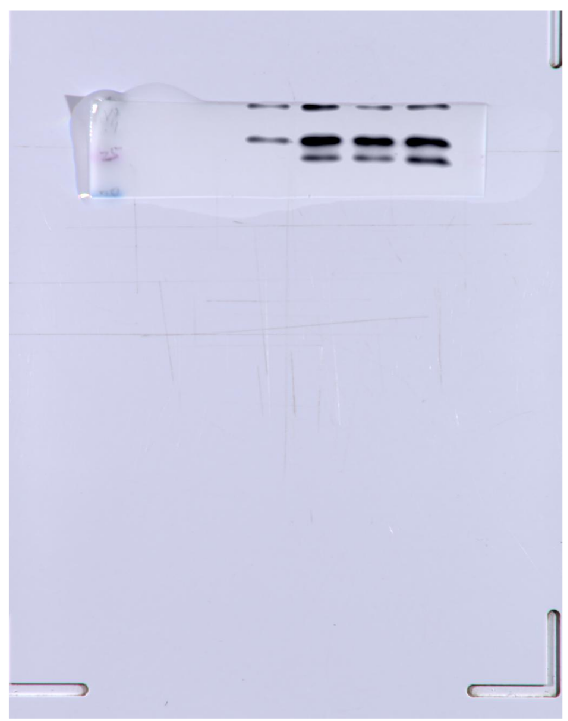

Supplement: Supplementary file 1 [file DataSheet_1.zip › original western blot image/Figure 1/A/GFP.tif]

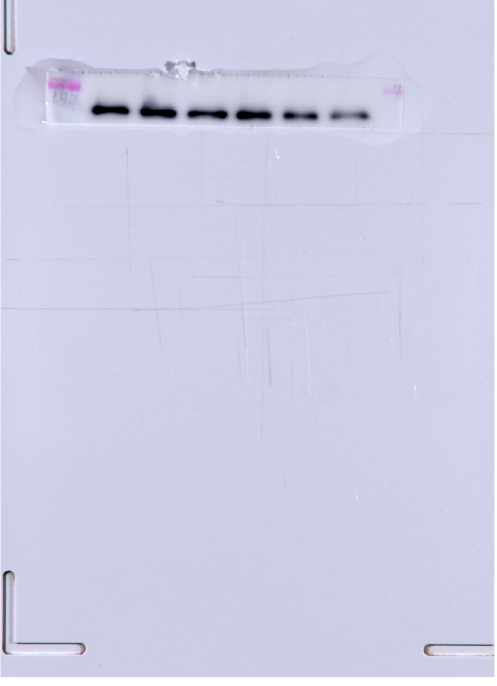

Supplement: Supplementary file 1 [file DataSheet_1.zip › original western blot image/Figure 1/A/HDAC8.tif]

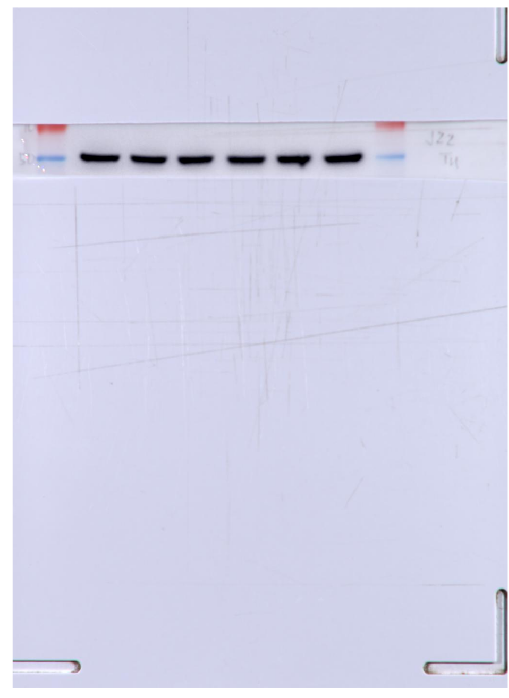

Supplement: Supplementary file 1 [file DataSheet_1.zip › original western blot image/Figure 1/A/βtubulin.tif]

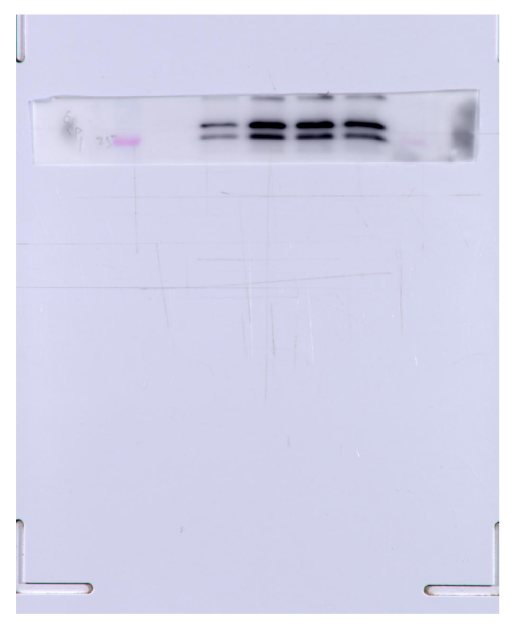

Supplement: Supplementary file 1 [file DataSheet_1.zip › original western blot image/Figure 1/B/GFP.tif]

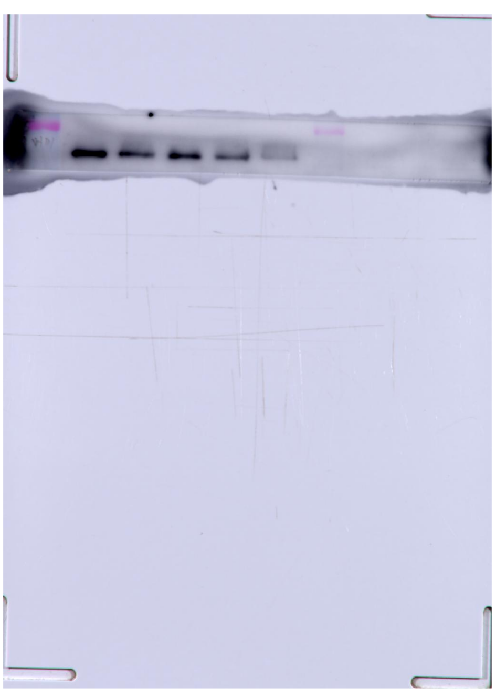

Supplement: Supplementary file 1 [file DataSheet_1.zip › original western blot image/Figure 1/B/HDAC8.tif]

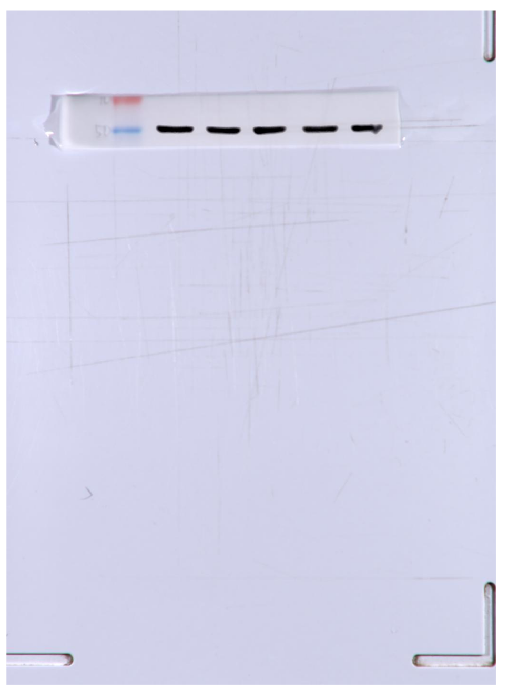

Supplement: Supplementary file 1 [file DataSheet_1.zip › original western blot image/Figure 1/B/βtubulin.tif]

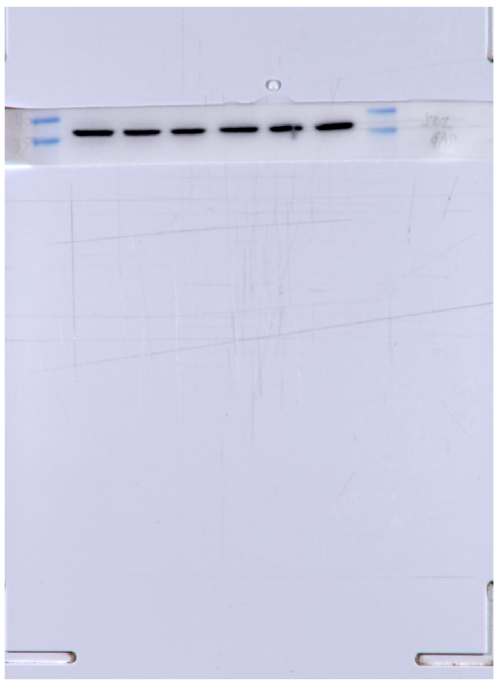

Supplement: Supplementary file 1 [file DataSheet_1.zip › original western blot image/Figure 1/C/GAPDH.tif]

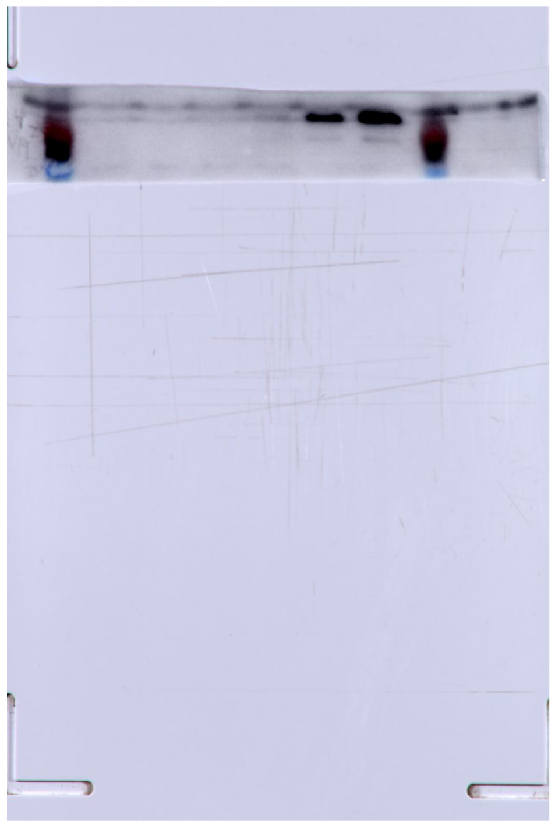

Supplement: Supplementary file 1 [file DataSheet_1.zip › original western blot image/Figure 1/C/GFP.tif]

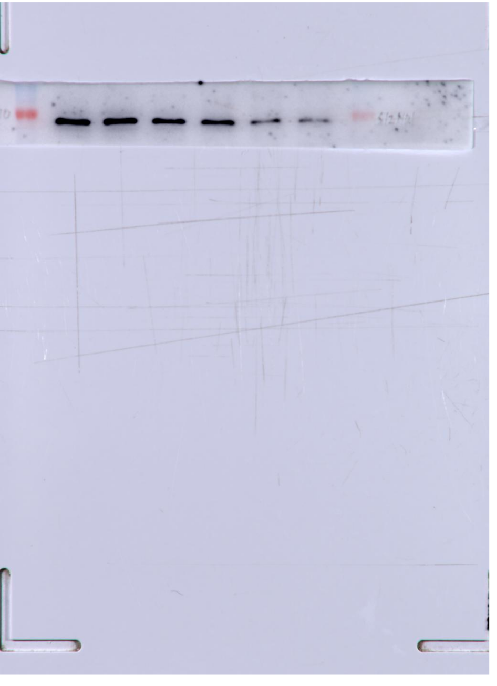

Supplement: Supplementary file 1 [file DataSheet_1.zip › original western blot image/Figure 1/C/HDAC8.tif]

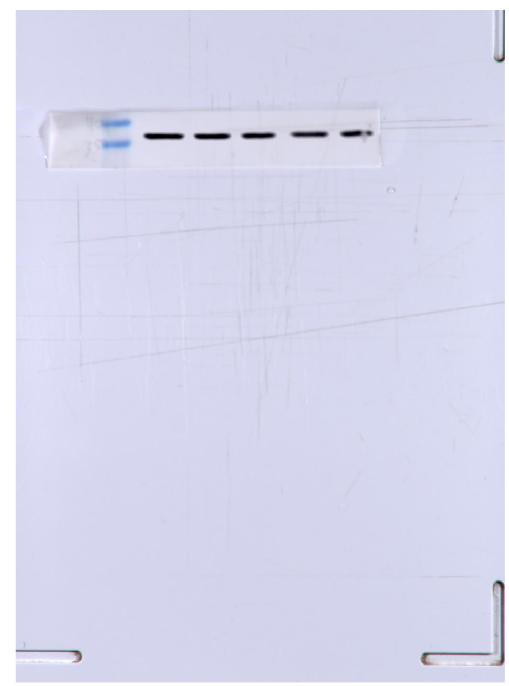

Supplement: Supplementary file 1 [file DataSheet_1.zip › original western blot image/Figure 1/D/GAPDH.tif]

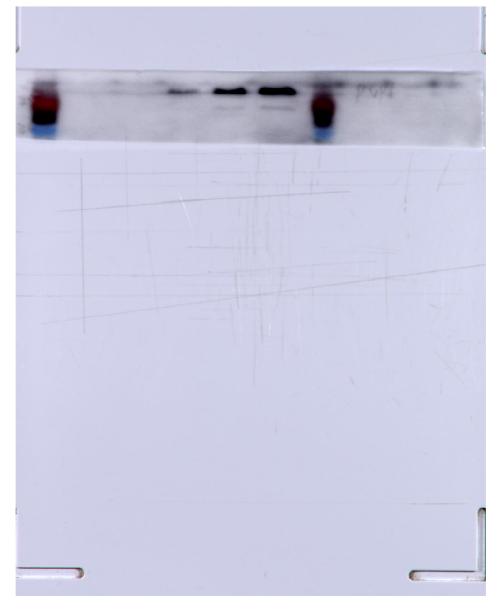

Supplement: Supplementary file 1 [file DataSheet_1.zip › original western blot image/Figure 1/D/GFP.tif]

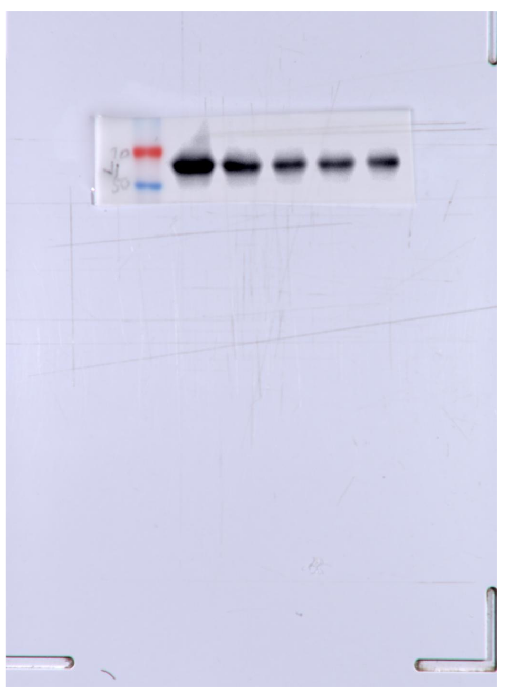

Supplement: Supplementary file 1 [file DataSheet_1.zip › original western blot image/Figure 1/D/HDAC8.tif]

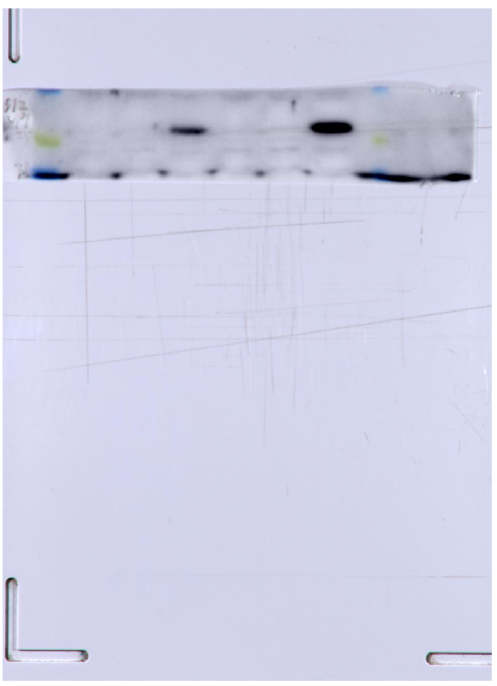

Supplement: Supplementary file 1 [file DataSheet_1.zip › original western blot image/Figure 2/A/GFP.tif]

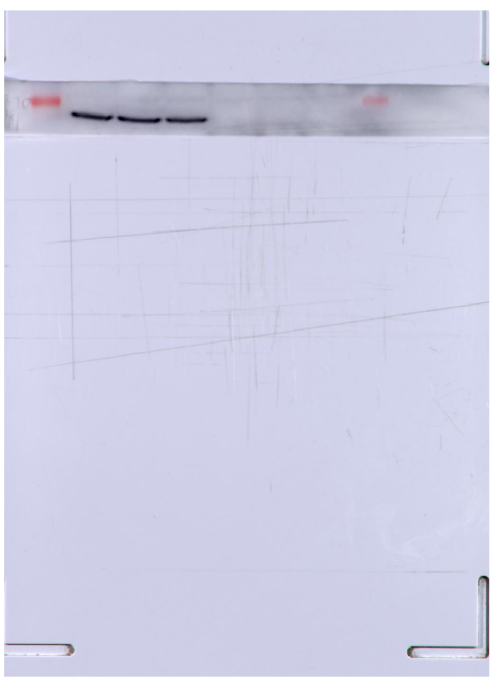

Supplement: Supplementary file 1 [file DataSheet_1.zip › original western blot image/Figure 2/A/HDAC8.tif]

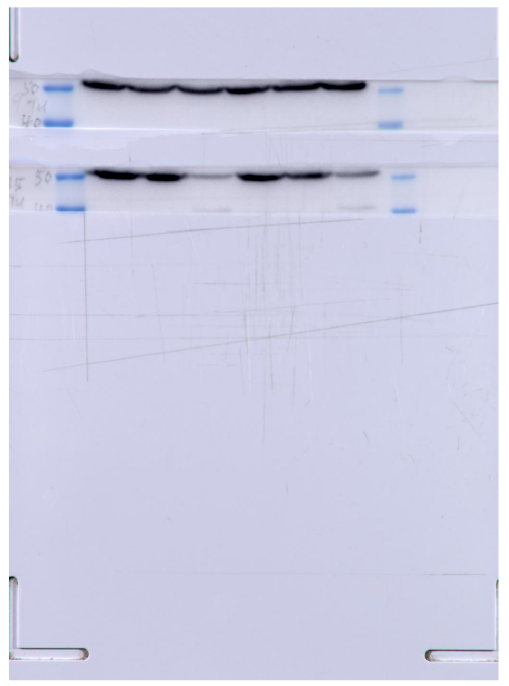

Supplement: Supplementary file 1 [file DataSheet_1.zip › original western blot image/Figure 2/A/βtubulin.tif]

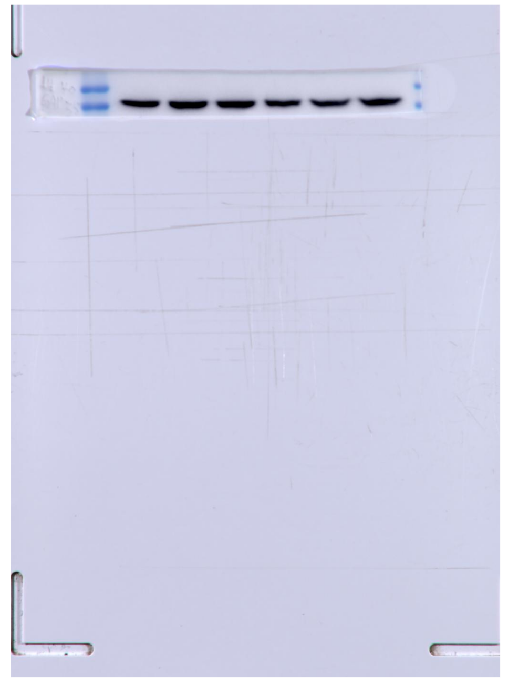

Supplement: Supplementary file 1 [file DataSheet_1.zip › original western blot image/Figure 2/B/GAPDH.tif]

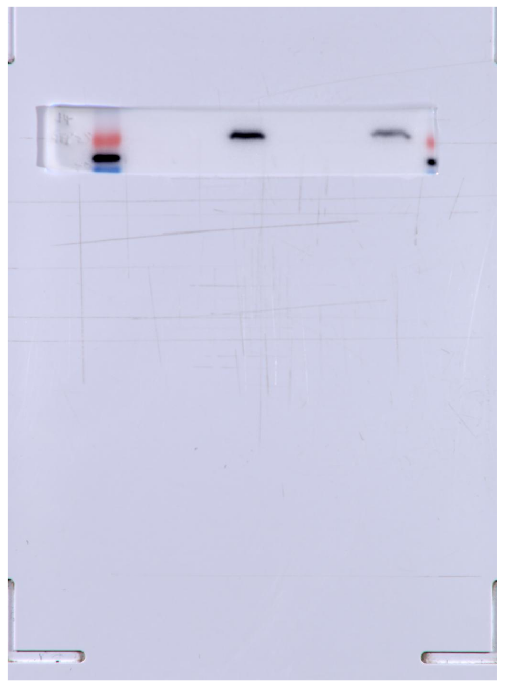

Supplement: Supplementary file 1 [file DataSheet_1.zip › original western blot image/Figure 2/B/GFP.tif]

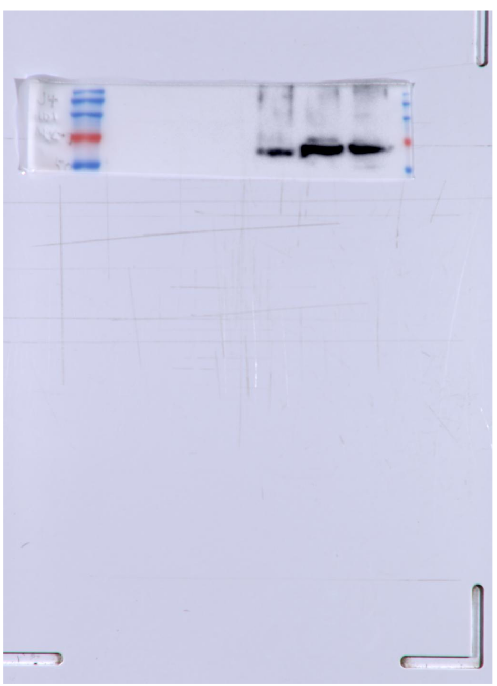

Supplement: Supplementary file 1 [file DataSheet_1.zip › original western blot image/Figure 2/B/MYC.tif]

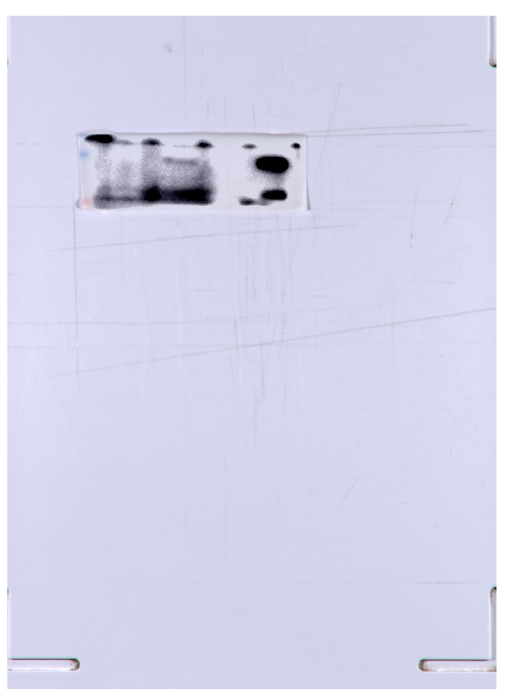

Supplement: Supplementary file 1 [file DataSheet_1.zip › original western blot image/Figure 2/C/GFP.tif]

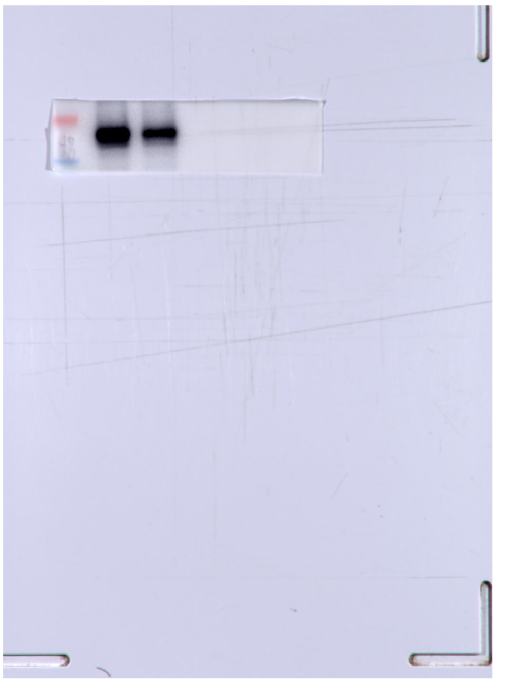

Supplement: Supplementary file 1 [file DataSheet_1.zip › original western blot image/Figure 2/C/HDAC8.tif]

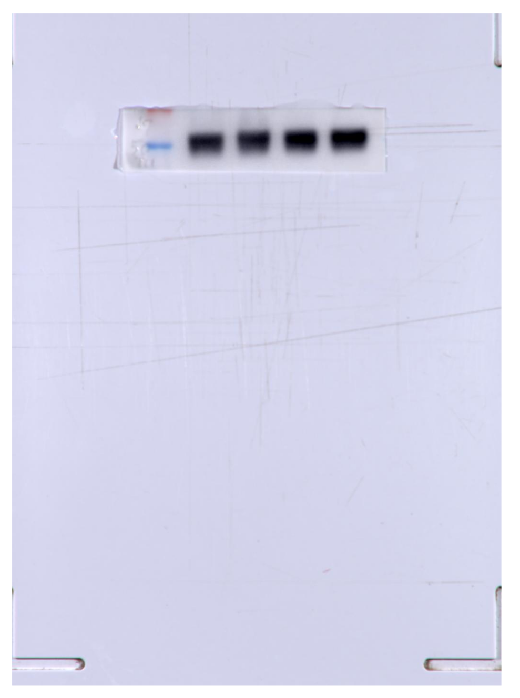

Supplement: Supplementary file 1 [file DataSheet_1.zip › original western blot image/Figure 2/C/βtubulin.tif]

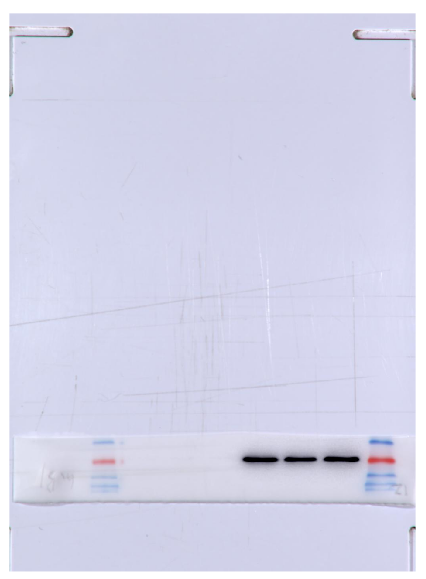

Supplement: Supplementary file 1 [file DataSheet_1.zip › original western blot image/Figure 2/D/FLAG.tif]

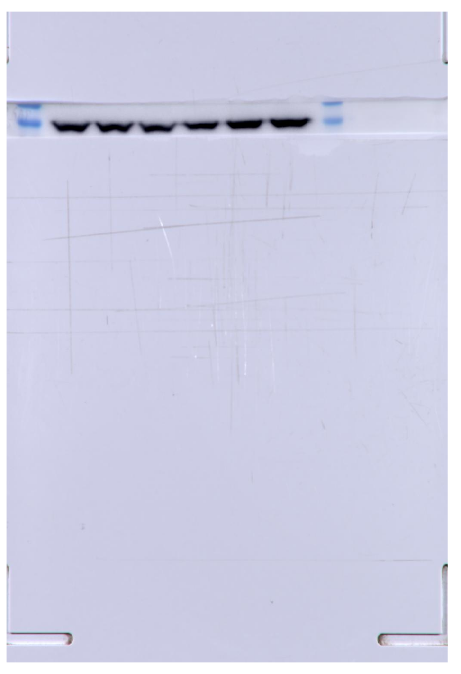

Supplement: Supplementary file 1 [file DataSheet_1.zip › original western blot image/Figure 2/D/GAPDH.tif]

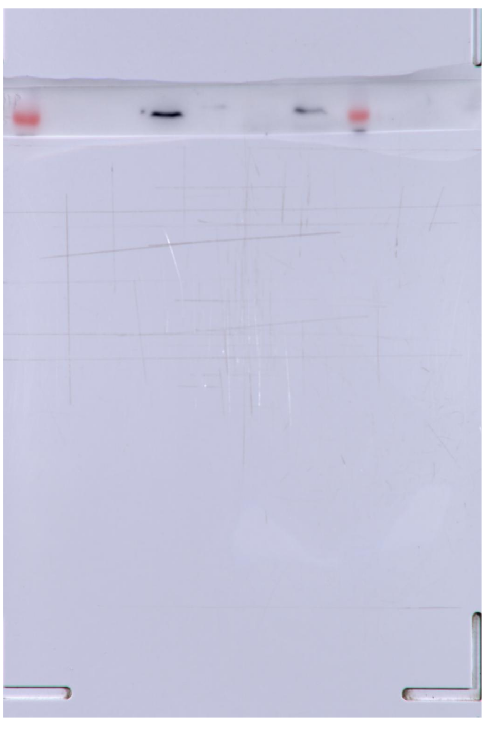

Supplement: Supplementary file 1 [file DataSheet_1.zip › original western blot image/Figure 2/D/GFP.tif]

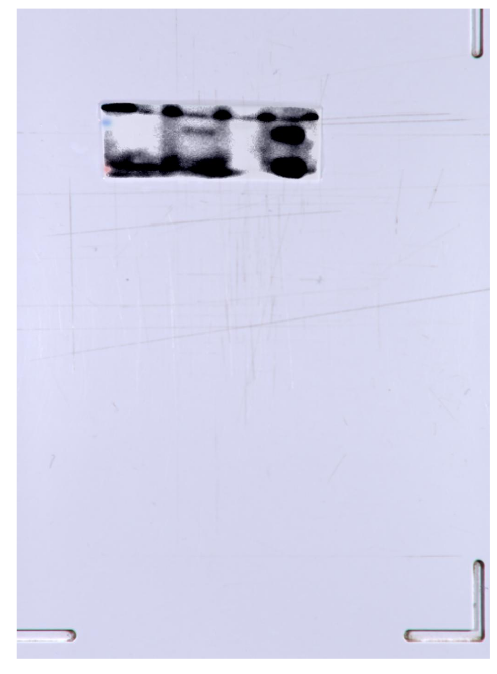

Supplement: Supplementary file 1 [file DataSheet_1.zip › original western blot image/Figure 3/E/GFP.tif]

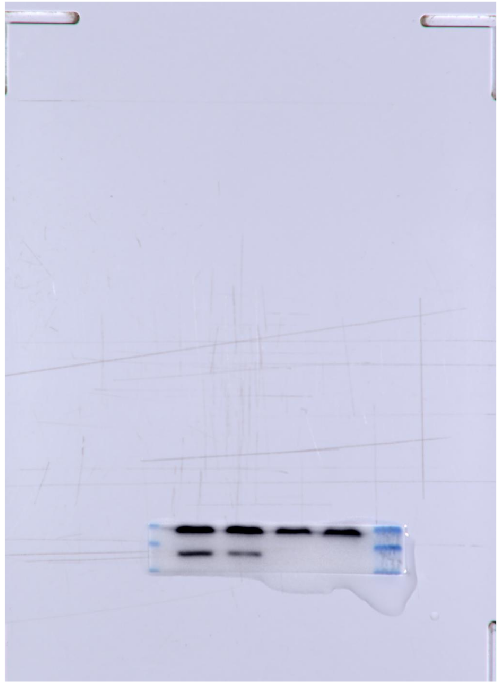

Supplement: Supplementary file 1 [file DataSheet_1.zip › original western blot image/Figure 3/E/HDAC8.tif]

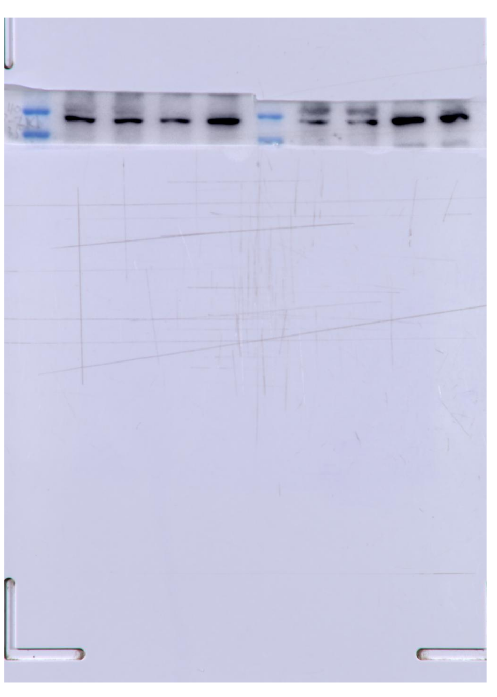

Supplement: Supplementary file 1 [file DataSheet_1.zip › original western blot image/Figure 3/E/IRF3.tif]

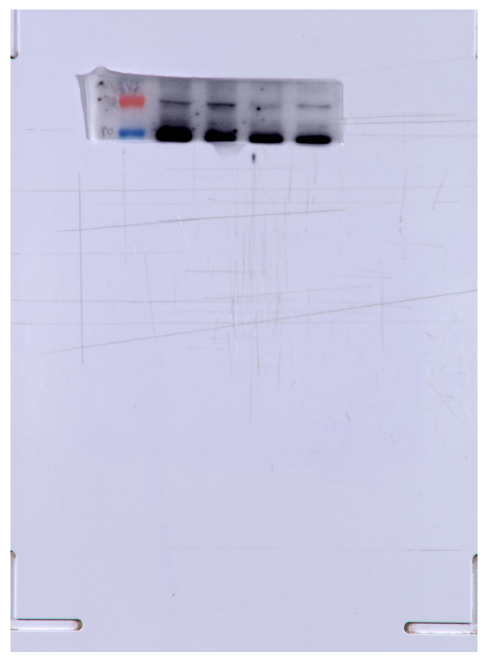

Supplement: Supplementary file 1 [file DataSheet_1.zip › original western blot image/Figure 3/E/MAVS.tif]

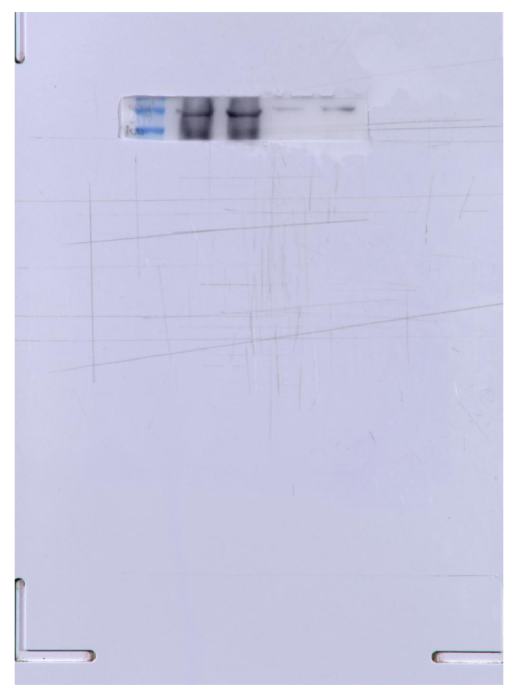

Supplement: Supplementary file 1 [file DataSheet_1.zip › original western blot image/Figure 3/E/RIG-I.tif]

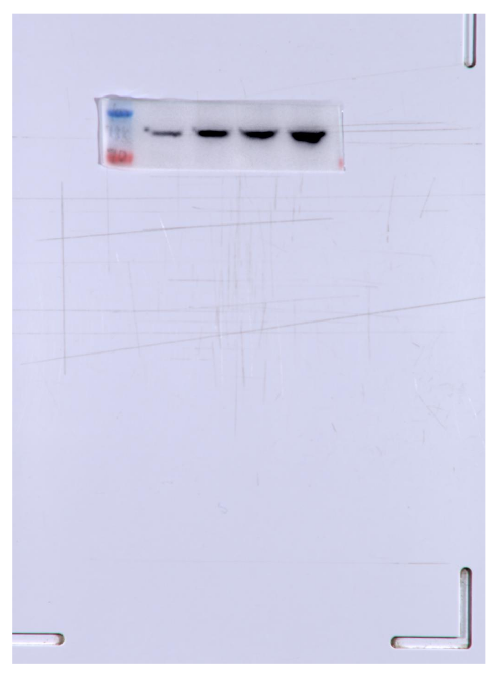

Supplement: Supplementary file 1 [file DataSheet_1.zip › original western blot image/Figure 3/E/TBK1.tif]

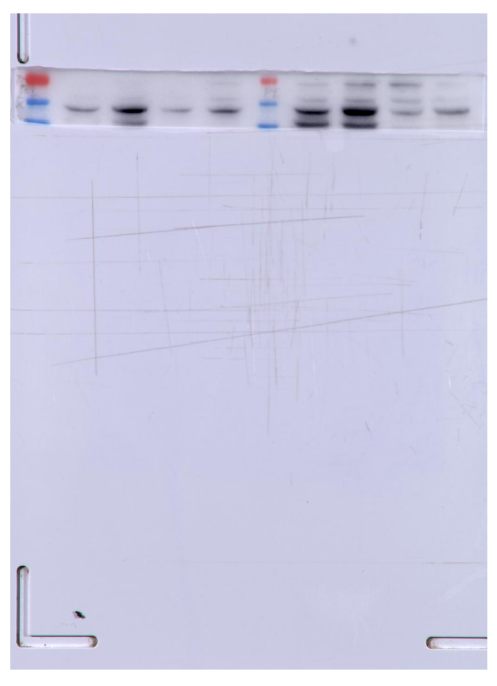

Supplement: Supplementary file 1 [file DataSheet_1.zip › original western blot image/Figure 3/E/p-IRF3.tif]

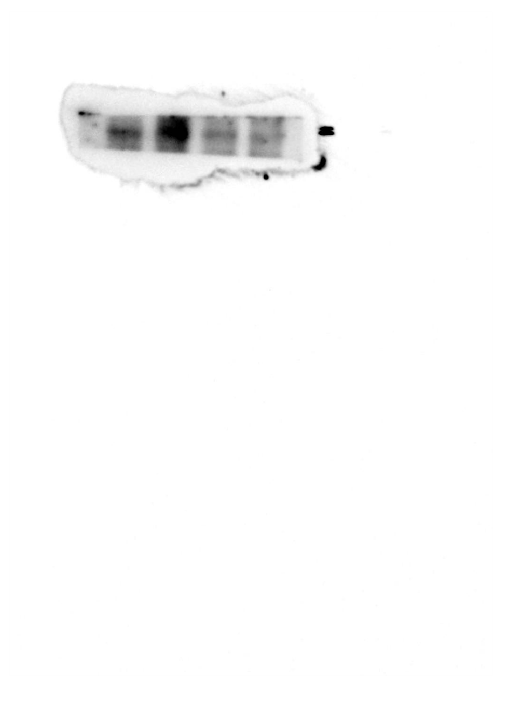

Supplement: Supplementary file 1 [file DataSheet_1.zip › original western blot image/Figure 3/E/p-TBK1.tif]

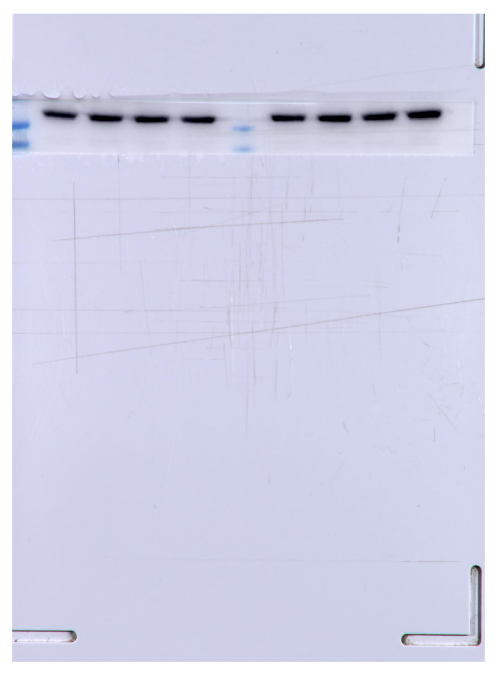

Supplement: Supplementary file 1 [file DataSheet_1.zip › original western blot image/Figure 3/E/βactin.tif]

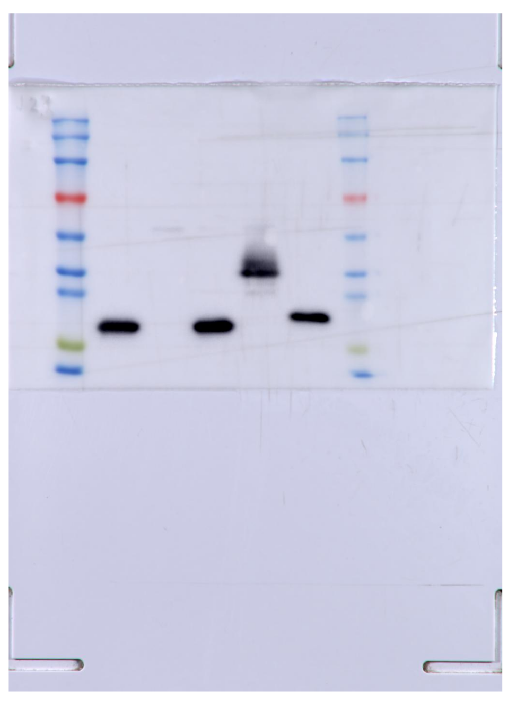

Supplement: Supplementary file 1 [file DataSheet_1.zip › original western blot image/Figure 4/A/IP/FLAG.tif]

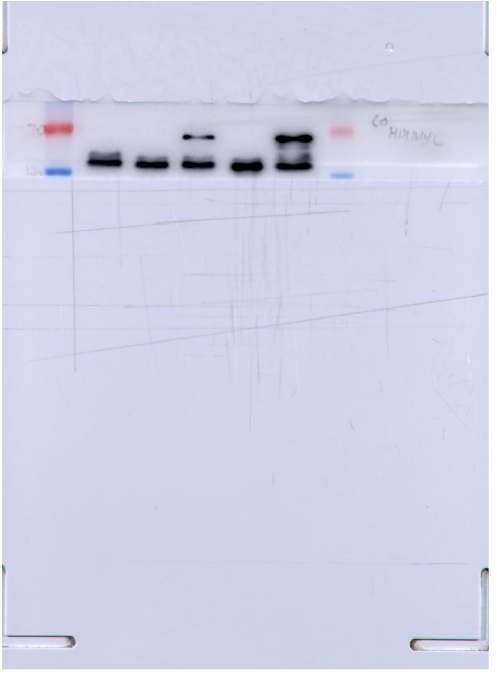

Supplement: Supplementary file 1 [file DataSheet_1.zip › original western blot image/Figure 4/A/IP/MYC.tif]

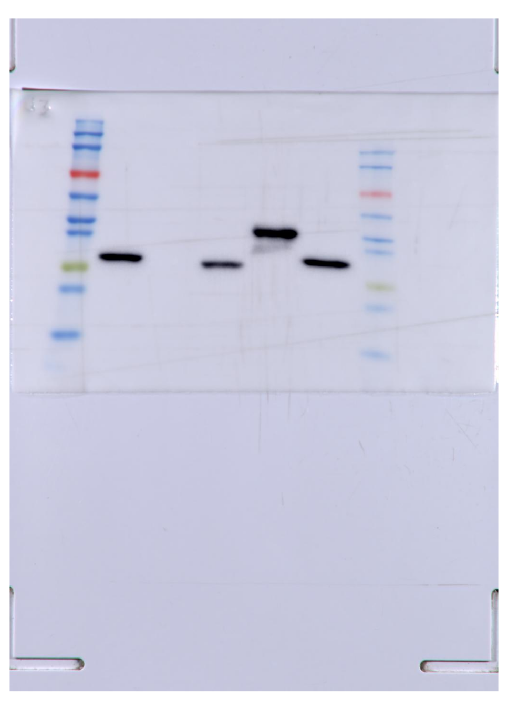

Supplement: Supplementary file 1 [file DataSheet_1.zip › original western blot image/Figure 4/A/WCL/FLAG.tif]

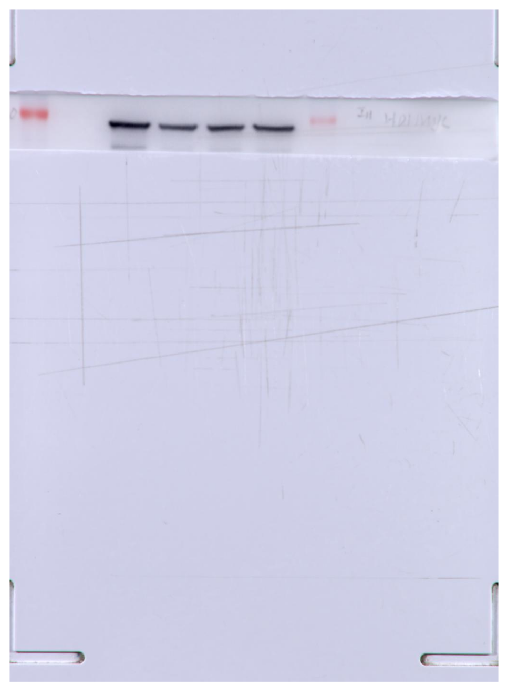

Supplement: Supplementary file 1 [file DataSheet_1.zip › original western blot image/Figure 4/A/WCL/MYC.tif]

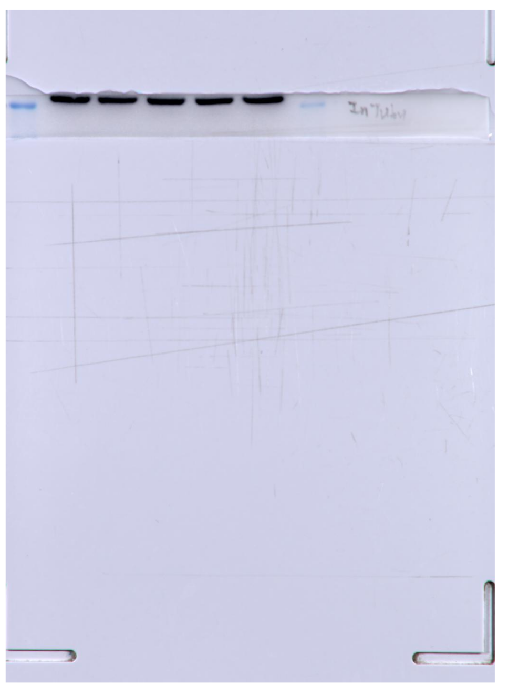

Supplement: Supplementary file 1 [file DataSheet_1.zip › original western blot image/Figure 4/A/WCL/βtubulin.tif]

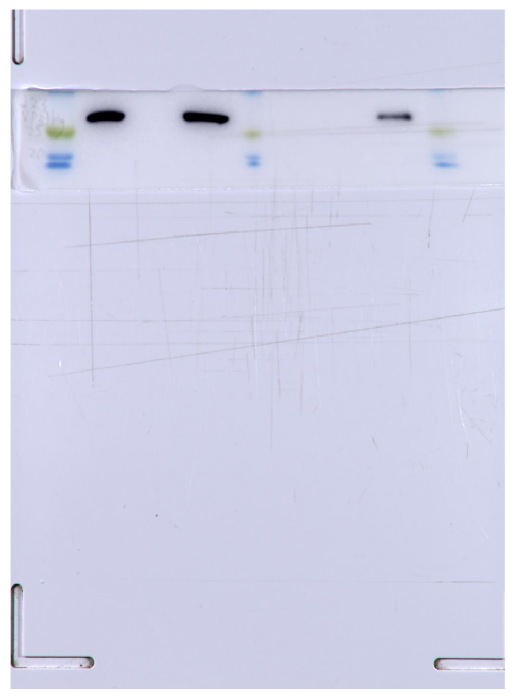

Supplement: Supplementary file 1 [file DataSheet_1.zip › original western blot image/Figure 4/B/IP/FLAG.tif]

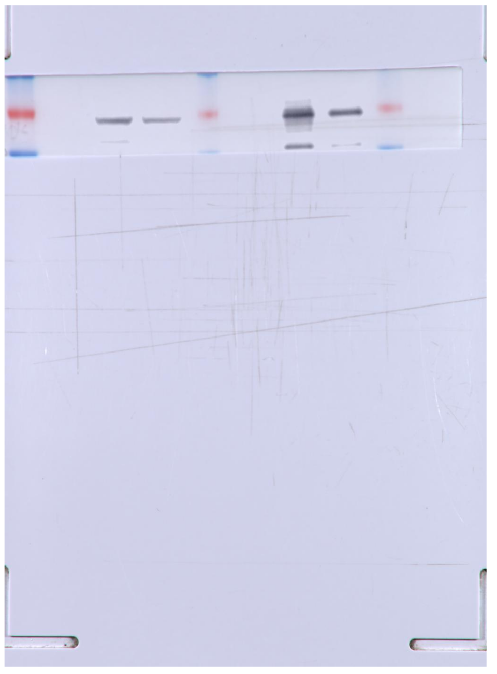

Supplement: Supplementary file 1 [file DataSheet_1.zip › original western blot image/Figure 4/B/IP/MYC.tif]

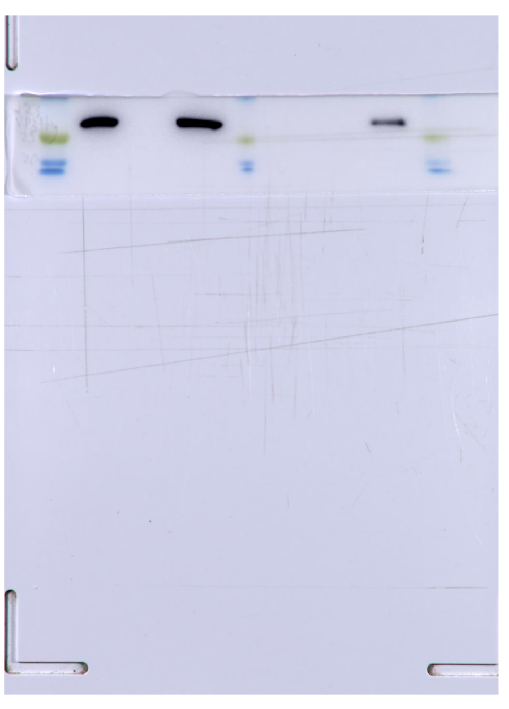

Supplement: Supplementary file 1 [file DataSheet_1.zip › original western blot image/Figure 4/B/WCL/FLAG.tif]

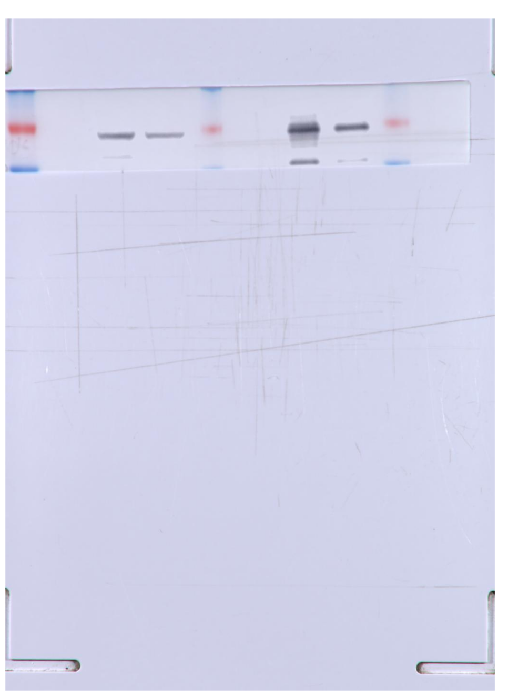

Supplement: Supplementary file 1 [file DataSheet_1.zip › original western blot image/Figure 4/B/WCL/MYC.tif]

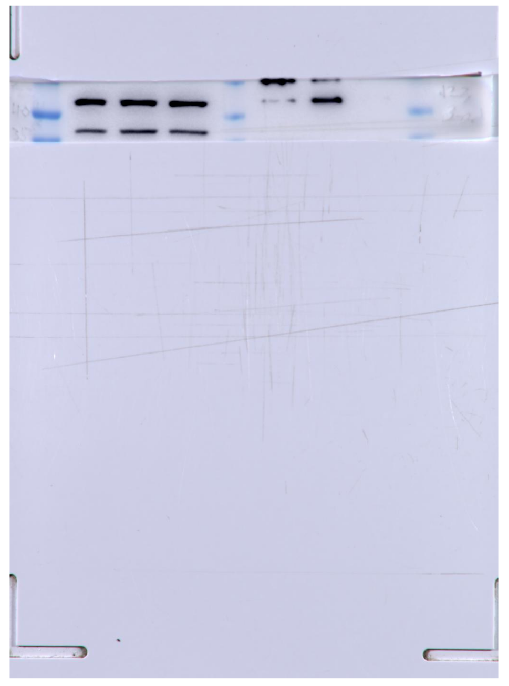

Supplement: Supplementary file 1 [file DataSheet_1.zip › original western blot image/Figure 4/B/WCL/βactin.tif]

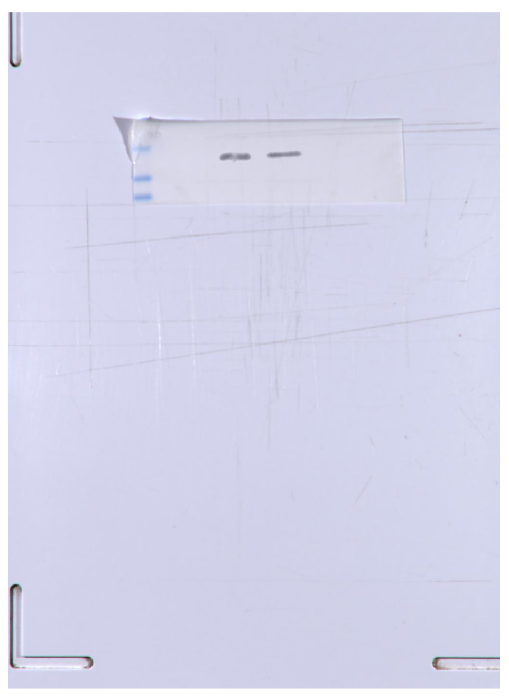

Supplement: Supplementary file 1 [file DataSheet_1.zip › original western blot image/Figure 4/D/IP/HA.tif]

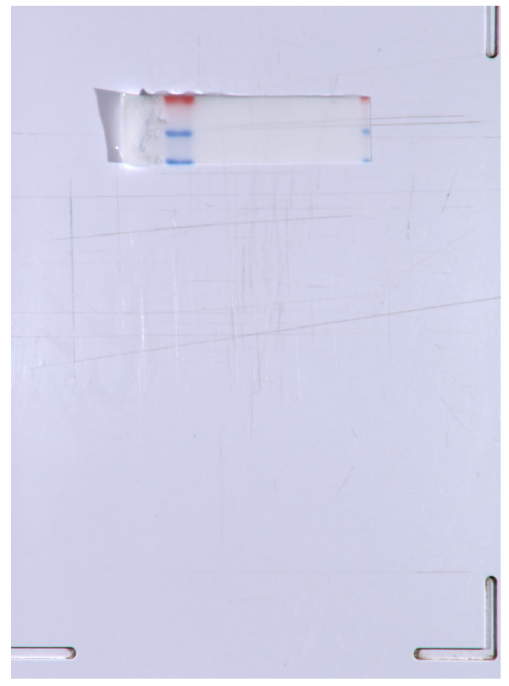

Supplement: Supplementary file 1 [file DataSheet_1.zip › original western blot image/Figure 4/D/IP/MYC.tif]

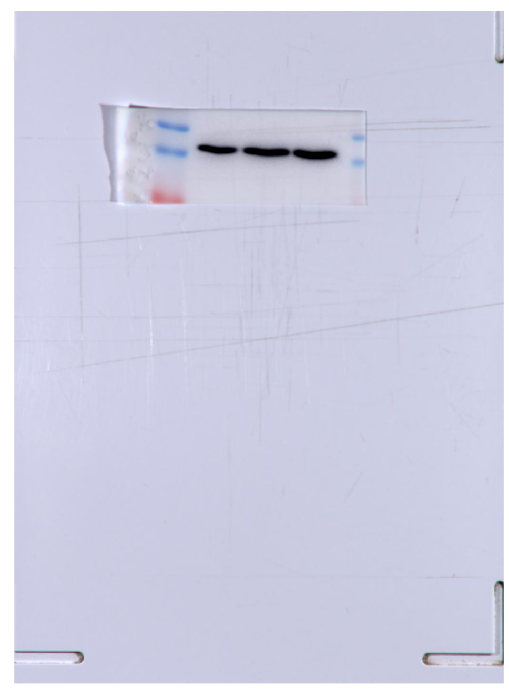

Supplement: Supplementary file 1 [file DataSheet_1.zip › original western blot image/Figure 4/D/WCL/GAPDH.tif]

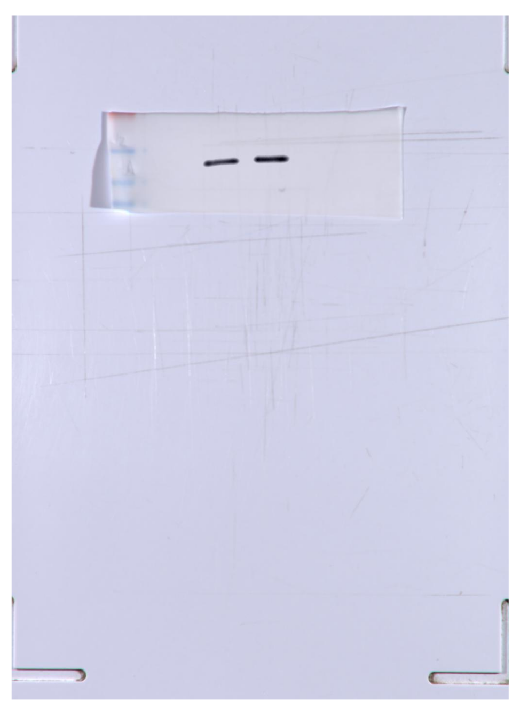

Supplement: Supplementary file 1 [file DataSheet_1.zip › original western blot image/Figure 4/D/WCL/HA.tif]

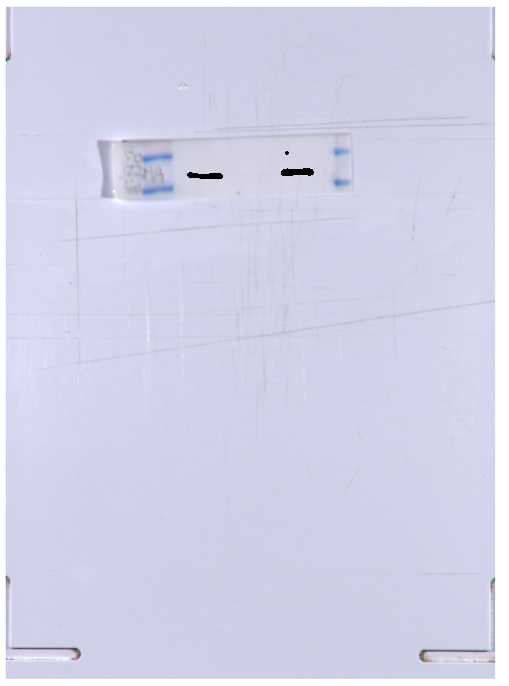

Supplement: Supplementary file 1 [file DataSheet_1.zip › original western blot image/Figure 4/D/WCL/MYC.tif]

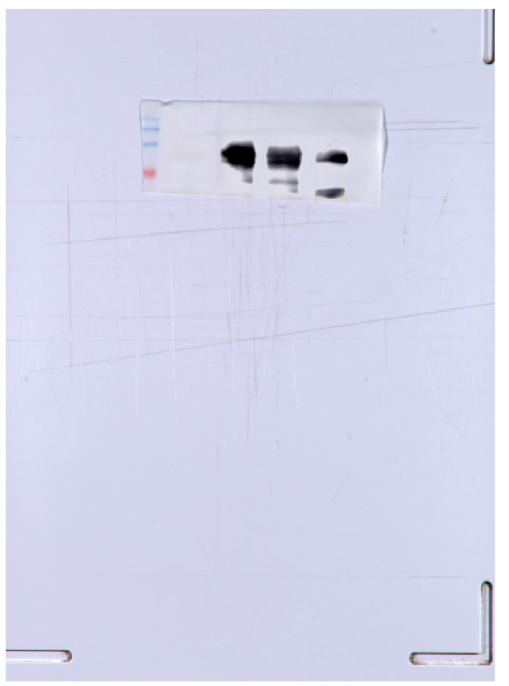

Supplement: Supplementary file 1 [file DataSheet_1.zip › original western blot image/Figure 4/E/IP/FLAG.tif]

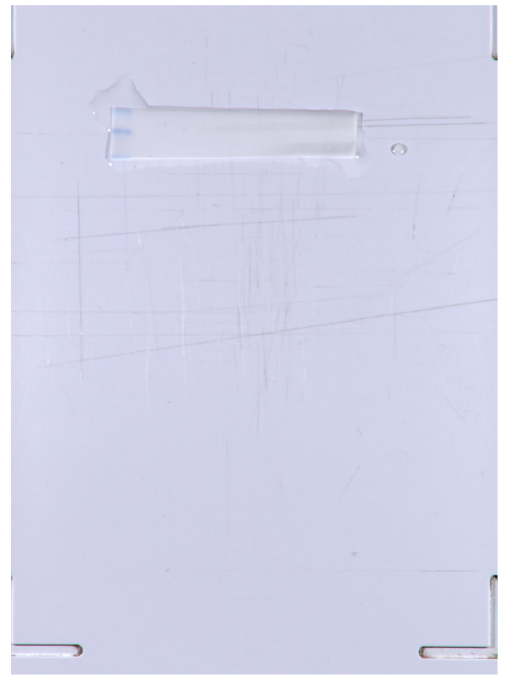

Supplement: Supplementary file 1 [file DataSheet_1.zip › original western blot image/Figure 4/E/IP/HA.tif]

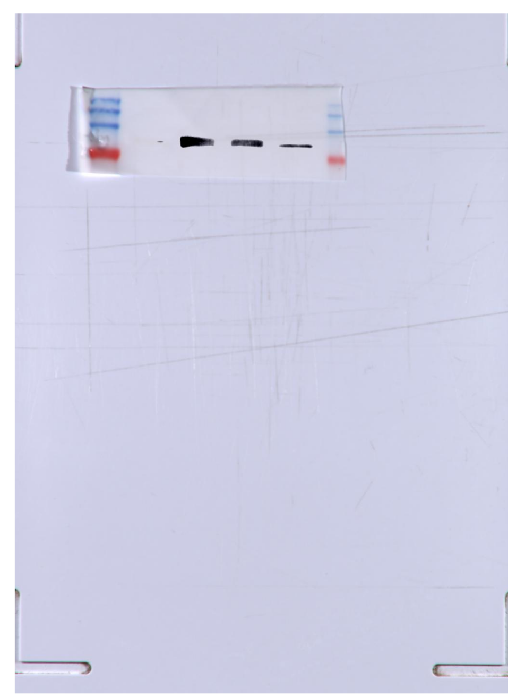

Supplement: Supplementary file 1 [file DataSheet_1.zip › original western blot image/Figure 4/E/WCL/FLAG.tif]

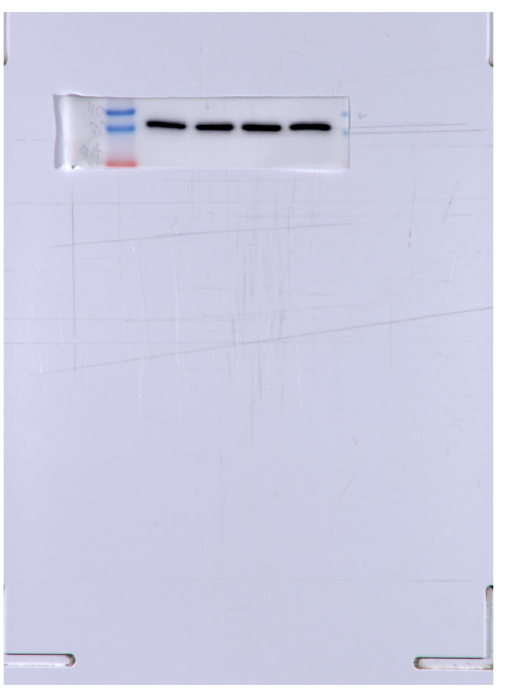

Supplement: Supplementary file 1 [file DataSheet_1.zip › original western blot image/Figure 4/E/WCL/GAPDH.tif]

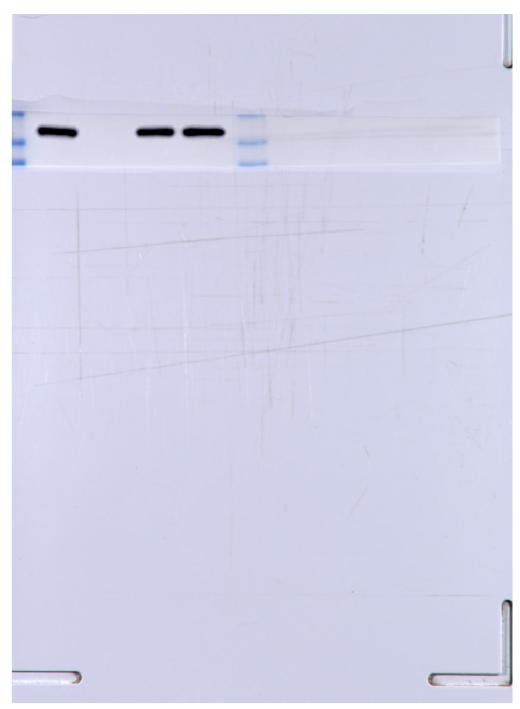

Supplement: Supplementary file 1 [file DataSheet_1.zip › original western blot image/Figure 4/E/WCL/HA.tif]

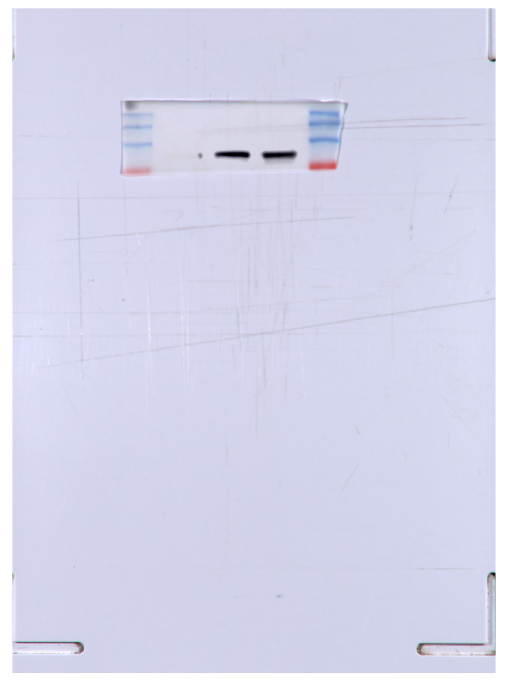

Supplement: Supplementary file 1 [file DataSheet_1.zip › original western blot image/Figure 4/F/IP/FLAG.tif]

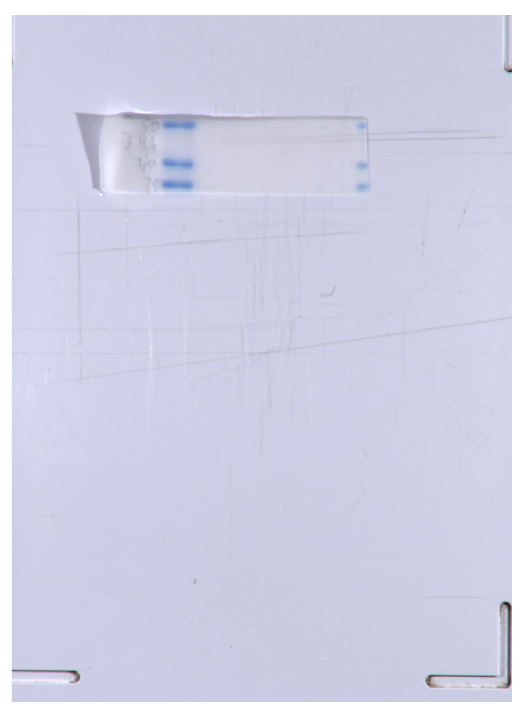

Supplement: Supplementary file 1 [file DataSheet_1.zip › original western blot image/Figure 4/F/IP/HA.tif]

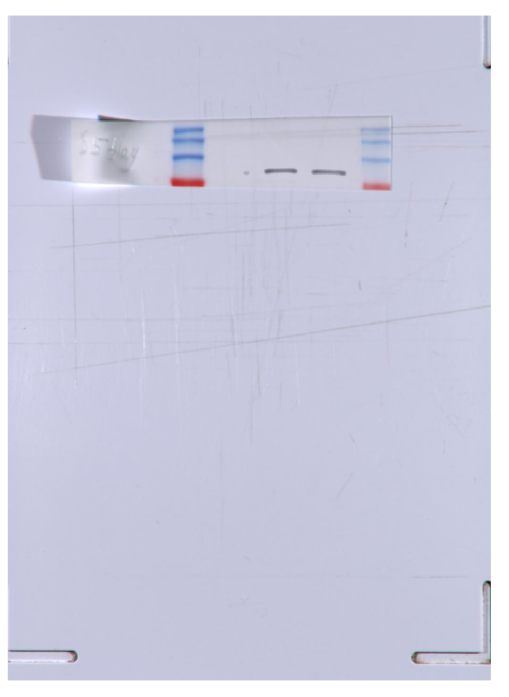

Supplement: Supplementary file 1 [file DataSheet_1.zip › original western blot image/Figure 4/F/WCL/FLAG.tif]

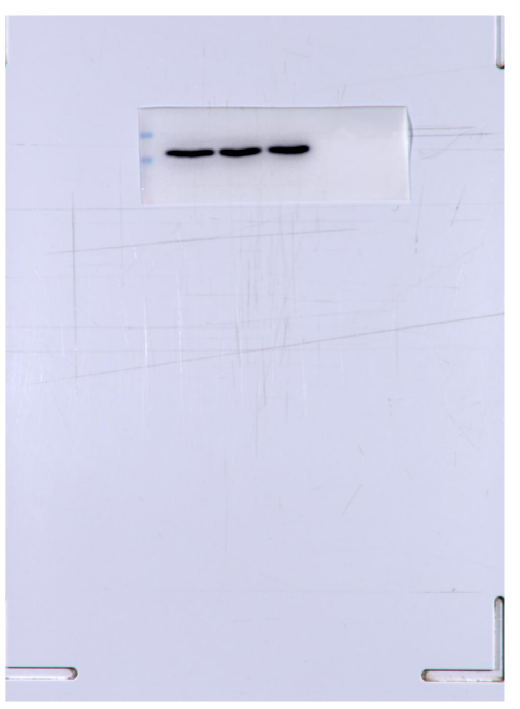

Supplement: Supplementary file 1 [file DataSheet_1.zip › original western blot image/Figure 4/F/WCL/GAPDH.tif]

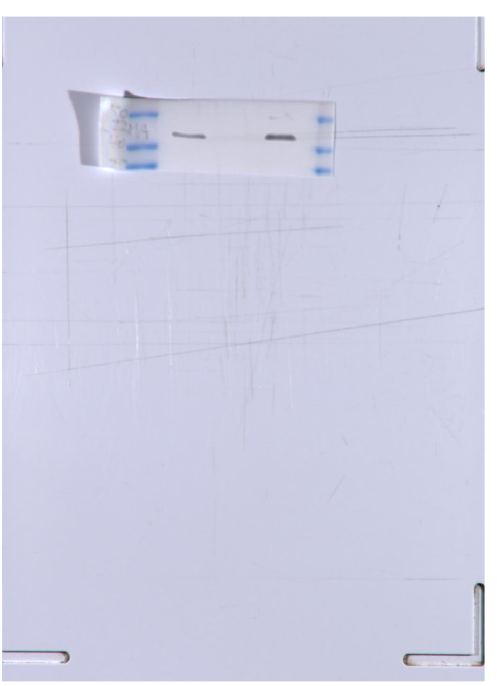

Supplement: Supplementary file 1 [file DataSheet_1.zip › original western blot image/Figure 4/F/WCL/HA.tif]

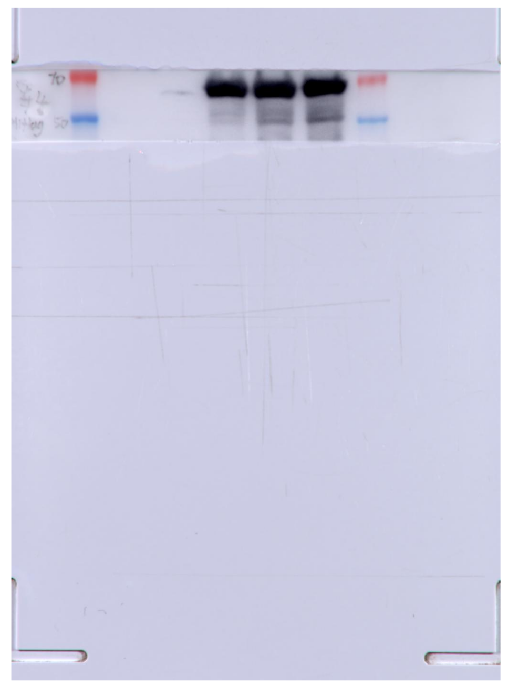

Supplement: Supplementary file 1 [file DataSheet_1.zip › original western blot image/Figure 5/A/FLAG.tif]

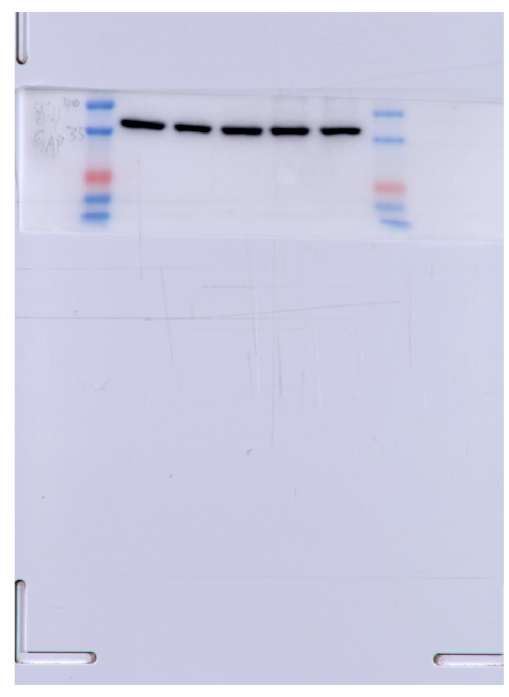

Supplement: Supplementary file 1 [file DataSheet_1.zip › original western blot image/Figure 5/A/GAPDH.tif]

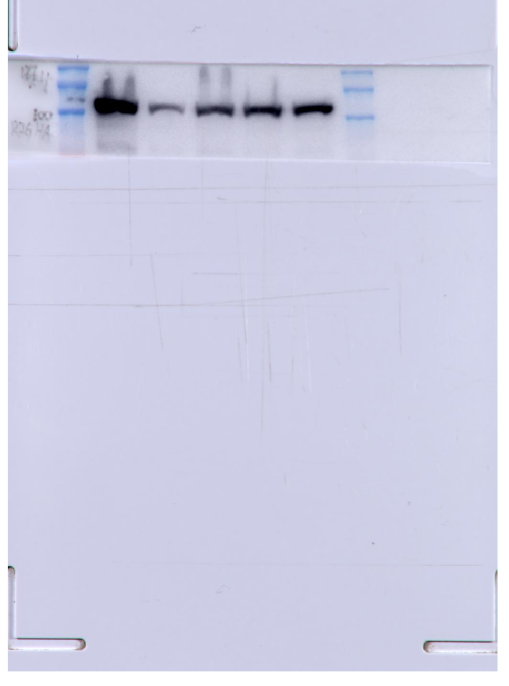

Supplement: Supplementary file 1 [file DataSheet_1.zip › original western blot image/Figure 5/A/HA.tif]

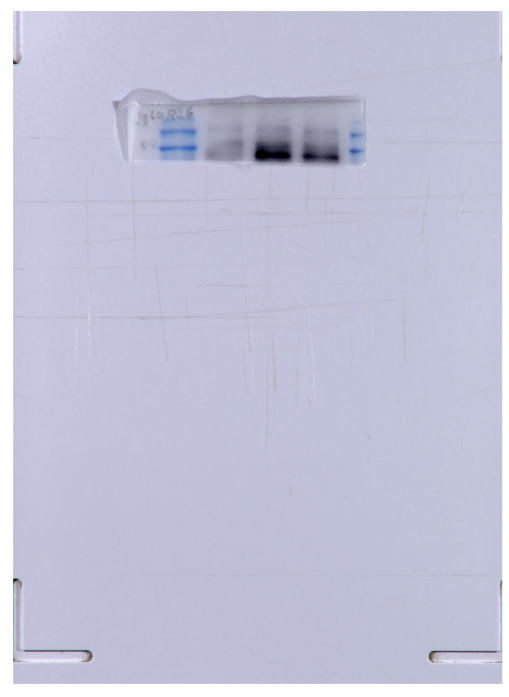

Supplement: Supplementary file 1 [file DataSheet_1.zip › original western blot image/Figure 5/B/IP/RIG-I.tif]

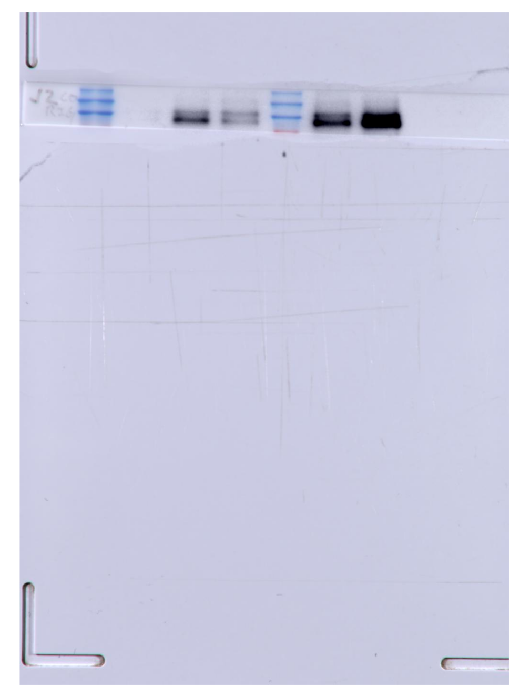

Supplement: Supplementary file 1 [file DataSheet_1.zip › original western blot image/Figure 5/B/IP/RIGI.tif]

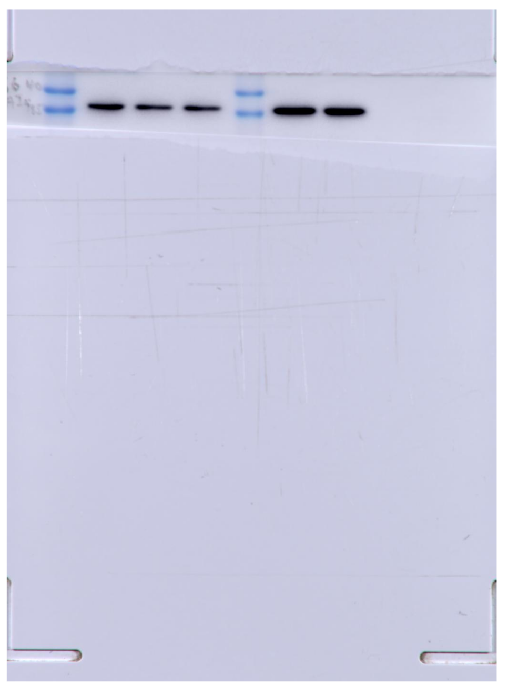

Supplement: Supplementary file 1 [file DataSheet_1.zip › original western blot image/Figure 5/B/WCL/GAPDH.tif]

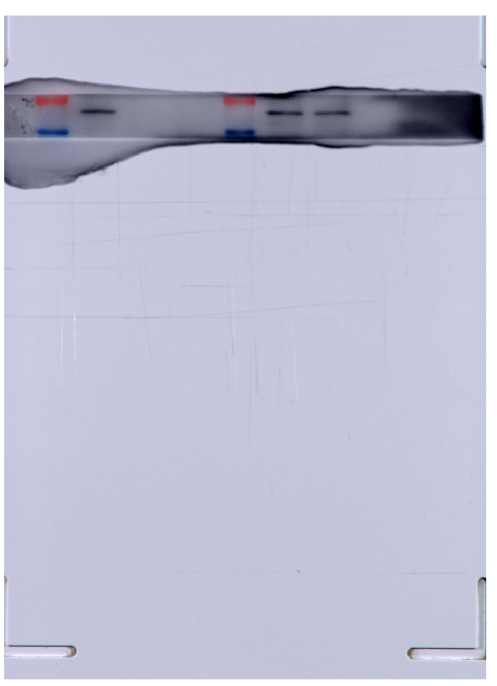

Supplement: Supplementary file 1 [file DataSheet_1.zip › original western blot image/Figure 5/B/WCL/HDAC8.tif]

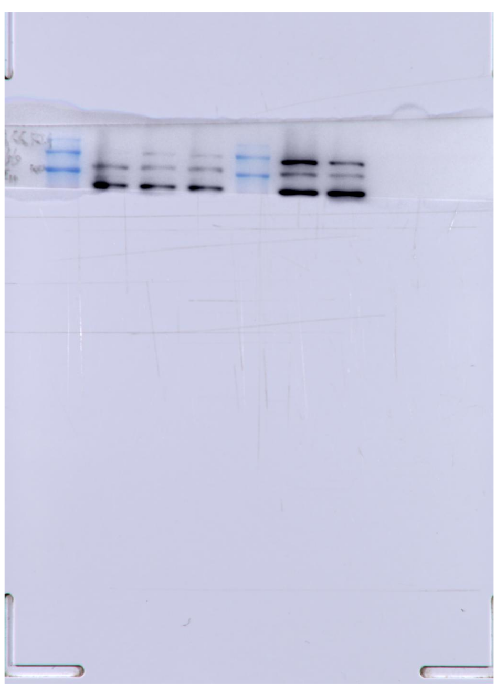

Supplement: Supplementary file 1 [file DataSheet_1.zip › original western blot image/Figure 5/B/WCL/RIG-I.tif]
