# Supplementary material for: Histone deacetylase 8 promotes innate antiviral immunity through deacetylation of RIG-I
Source: Front Cell Infect Microbiol. 2024 Jul 5;14:1415695. doi: 10.3389/fcimb.2024.1415695 (PMC11257846; doi:10.3389/fcimb.2024.1415695)
Supplement: Supplementary file 2 [file DataSheet_2.docx]

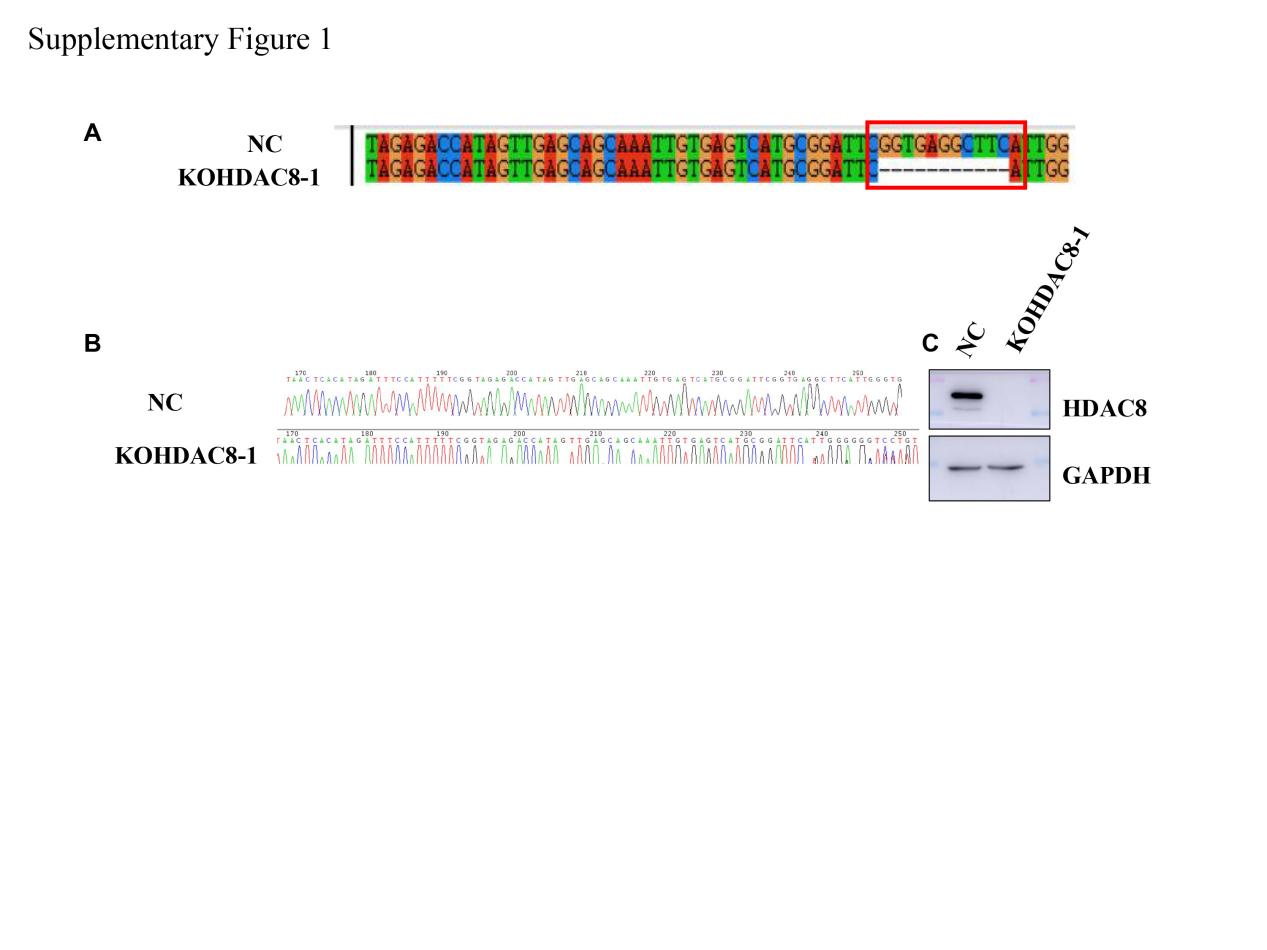


Figure S1 Confirmation of the establishment of HDAC8 knockout 293T cell lines. **(A)** Alignment of the NC (Negative control, cells transfected with empty vector and selected by puromycin), HDAC8-KO1, and HDAC8 cDNA sequences using Clustalx software. The red box indicates the sgRNA sequence and the mutations in the PAM motif. **(B)** Chromas software was used to confirm the sequencing peaks of PCR products of KOHDAC8-NC and HDAC8-KO1. **(C)** The expression of HDAC8 was detected by Western blot with HDAC8 antibody, and GAPDH was used as a control to show the even loading of samples.


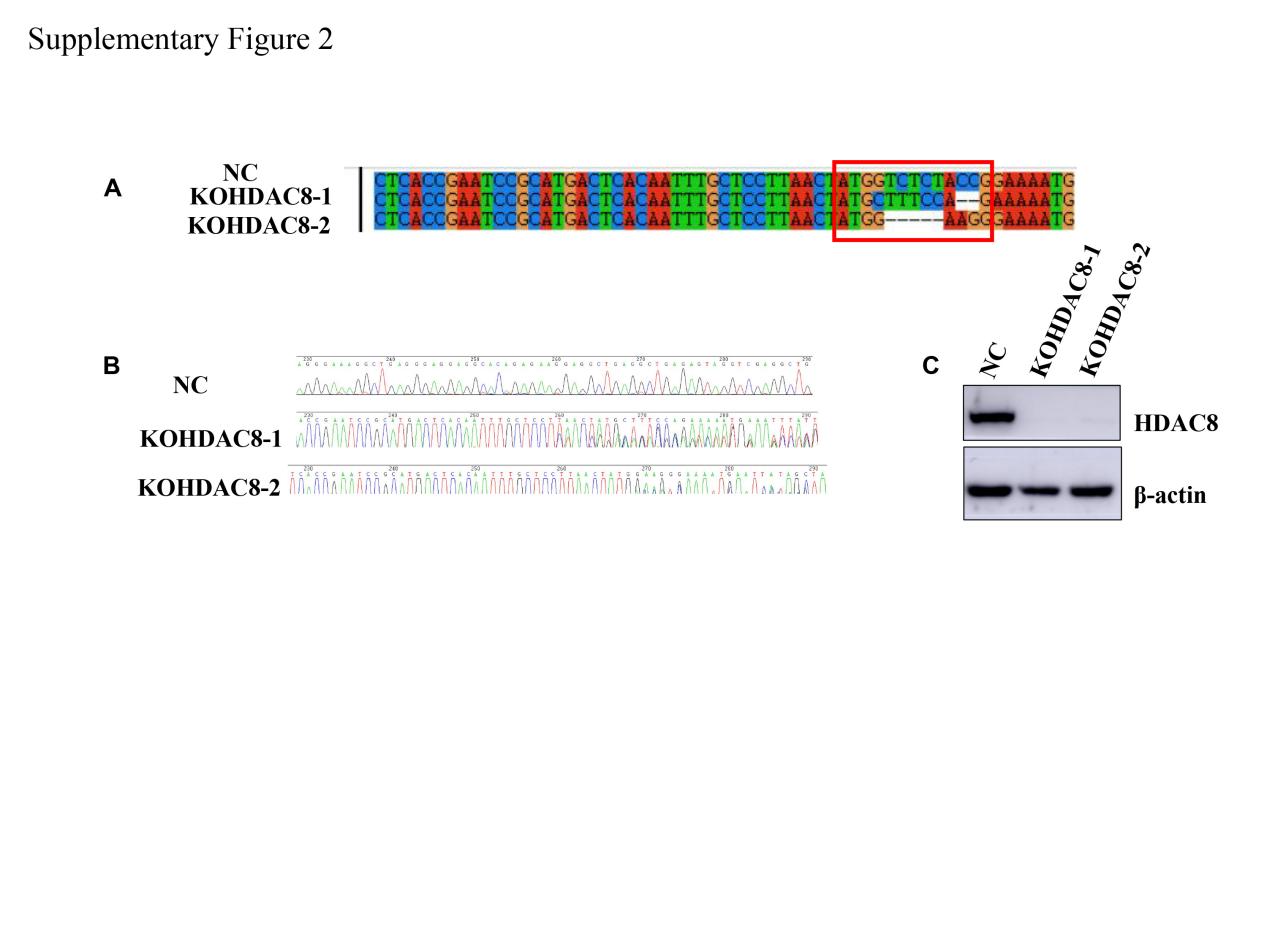


Figure S2 Confirmation of the establishment of HDAC8 knockout HeLa cell lines. **(A)** Alignment of the NC (Negative control, cells transfected with empty vector and selected by puromycin), HDAC8-KO1, HDAC8-KO2, and HDAC8 cDNA sequences using Clustalx software. The red box indicates the sgRNA sequence and the mutations in the PAM motif. **(B)** Chromas software was used to confirm the sequencing peaks of PCR products of KOHDAC8-NC, HDAC8-KO1, and HDAC8-KO2. **(C)** The expression of HDAC8 was detected by Western blot with HDAC8 antibody, and GAPDH was used as a control to show the even loading of samples.
